# Supplementary material for: Use of surveys to evaluate an integrated oral cholera vaccine campaign in response to a cholera outbreak in Hoima district, Uganda
Source: BMJ Open. 2020 Dec 10;10(12):e038464. doi: 10.1136/bmjopen-2020-038464 (PMC7733212; doi:10.1136/bmjopen-2020-038464)
Supplement: Author's manuscript [file bmjopen-2020-038464.draft_revisions.pdf]

BMJ Open is committed to open peer review. As part of this commitment we make the peer review history of every article we publish publicly available.

When an article is published we post the peer reviewers' comments and the authors' responses online. We also post the versions of the paper that were used during peer review. These are the versions that the peer review comments apply to.

The versions of the paper that follow are the versions that were submitted during the peer review process. They are not the versions of record or the final published versions. They should not be cited or distributed as the published version of this manuscript.

BMJ Open is an open access journal and the full, final, typeset and author-corrected version of record of the manuscript is available on our site with no access controls, subscription charges or pay-per-view fees (http://bmjopen.bmj.com).

If you have any questions on BMJ Open's open peer review process please email info.bmjopen@bmj.com

BMJ Open

Lessons and challenges of implementing an integrated oral cholera vaccine and WaSH response to a cholera epidemic in Hoima district, Uganda

Journal:	*BMJ Open*
Manuscript ID	bmjopen-2020-038464
Article Type:	Original research
Date Submitted by the Author:	11-Mar-2020
Complete List of Authors:	Bwire, Godfrey; Republic of Uganda Ministry of Health, Community Health<br>Roskosky, Mellisa; Johns Hopkins University Bloomberg School of Public Health, International Health<br>Ballard, Anne; Johns Hopkins University Bloomberg School of Public Health, Center for Communications Programs<br>Brooks, W; Johns Hopkins University Bloomberg School of Public Health, International Health<br>Alfred, Okello; Makerere University College of Health Sciences<br>Rafael, Florentina; World Health Organization, Infectious Hazard Management<br>Ampaire, Immaculate; Republic of Uganda Ministry of Health, Expanded Program on Immunization<br>Orach, Christopher; Makerere University College of Health Sciences<br>Sack, David; Johns Hopkins University Bloomberg School of Public Health, International Health
Keywords:	Gastrointestinal infections < GASTROENTEROLOGY, International health services < HEALTH SERVICES ADMINISTRATION & MANAGEMENT, Epidemiology < INFECTIOUS DISEASES, Infection control < INFECTIOUS DISEASES, Public health < INFECTIOUS DISEASES, Tropical medicine < INFECTIOUS DISEASES

SCHOLARONE™
Manuscripts

I, the Submitting Author has the right to grant and does grant on behalf of all authors of the Work (as defined in the below author licence), an exclusive licence and/or a non-exclusive licence for contributions from authors who are: i) UK Crown employees; ii) where BMJ has agreed a CC-BY licence shall apply, and/or iii) in accordance with the terms applicable for US Federal Government officers or employees acting as part of their official duties; on a worldwide, perpetual, irrevocable, royalty-free basis to BMJ Publishing Group Ltd ("BMJ") its licensees and where the relevant Journal is co-owned by BMJ to the co-owners of the Journal, to publish the Work in this journal and any other BMJ products and to exploit all rights, as set out in our licence.

The Submitting Author accepts and understands that any supply made under these terms is made by BMJ to the Submitting Author unless you are acting as an employee on behalf of your employer or a postgraduate student of an affiliated institution which is paying any applicable article publishing charge ("APC") for Open Access articles. Where the Submitting Author wishes to make the Work available on an Open Access basis (and intends to pay the relevant APC), the terms of reuse of such Open Access shall be governed by a Creative Commons licence – details of these licences and which Creative Commons licence will apply to this Work are set out in our licence referred to above.

Other than as permitted in any relevant BMJ Author's Self Archiving Policies, I confirm this Work has not been accepted for publication elsewhere, is not being considered for publication elsewhere and does not duplicate material already published. I confirm all authors consent to publication of this Work and authorise the granting of this licence.

For peer review only

Lessons and challenges of implementing an integrated oral cholera vaccine and WaSH response to a cholera epidemic in Hoima district, Uganda

Godfrey Bwire[1,] Mellisa Roskosky[2], Anne Ballard Sara[2], W. Abdullah Brooks[2], Alfred Okello[3], Florentina Rafael [4], Immaculate Ampeire[5], Christopher Garimoi Orach[3], David A. Sack[2].

Authors' affiliations:

1. Ministry of Health, Department of Community Health, Kampala Uganda.
2. Johns Hopkins Bloomberg School of Public Health, Department of International Health, Baltimore, Maryland USA.
3. Makerere University, College of Health Sciences, Kampala, Uganda.
4. World Health Organization, Department of Infectious Hazard Management, Geneva, Switzerland.
5. Ministry of Health, Uganda National Expanded Program on Immunization

*Corresponding author.

Professor David A. Sack, M, D.
Johns Hopkins Bloomberg School of Public Health
615 North Wolfe Street
Baltimore, Maryland 21205 USA
Phone +1 443 287 8795
Email: dsack1@jhu.edu

Word count of title page: 134

Word count (Excluding abstract): 3677

Abstract word count: 314

Key words: cholera, vaccination, Uganda, monitoring, coverage, outbreak, Hoima, oral cholera vaccine, Vaccine campaign

For peer review only - http://bmjopen.bmj.com/site/about/guidelines.xhtml

ABSTRACT

Background. Cholera is preventable and treatable through application of surveillance, case management, water, sanitation and hygiene (WaSH) complemented by oral cholera vaccine (OCV). Vaccine was first introduced in Uganda during an outbreak in Hoima district in May - June 2018. Since OCV was new for Uganda, documentation of this campaign provided important lessons for future OCV campaigns in Uganda and elsewhere.

Methods. Surveys conducted during and post campaign consisted of two sub-studies. Sub-study one assessed the knowledge and practices of OCV staff who implemented the OCV campaign. Sub study two used a two-stage cluster random sampling technique to select 31 villages (clusters) from which 4 – 7 households were randomly selected and interviewed to assess vaccine coverage, the community's knowledge and practice of cholera prevention and the participant's understanding of OCV.

Results. In sub-study 1, most staff (93%) were knowledgeable about cholera control; however, 29% did not clearly understand detecting and managing adverse events following immunization (AEFI). In sub-study two, 209 households (1,259 individuals) were surveyed, of whom 1178 (93%) reported receiving at least one OCV dose and 986 (78%) reported receiving two doses. Among vaccinated individuals, minor complaints were reported by 71 persons (5.6%). Individuals with 'some' education (primary or secondary school) were more knowledgeable regarding the required OCV doses compared to non-educated (p-value = 0.04). Factors negatively associated with campaign implementation included community sensitisation time, staff payment and problems with field transport. Although the campaign was carried out quickly, the outbreak was over before the campaign started.

Conclusion. The campaign achieved high OCV coverage, but the surveys provided insights for improvement. For greater vaccine coverage, more effort is needed for community sensitisation, and additional resources for staff transportation and timely payment is required. Pre and post-test assessment of staff training can identify and address knowledge and skill gaps.

For peer review only - http://bmjopen.bmj.com/site/about/guidelines.xhtml

Strengths and limitations of this study

- This is an evaluation of the first oral cholera vaccine (OCV) campaign in Uganda
- Coverage rates were high, indicating a successful campaign
- Evaluation of the vaccination staff immediately following each round identified ways to improve performance in the next round.
- Communities readily accepted OCV, but some were not sure of the timing of the second dose
- Improvements were needed in the program to detect and manage potential adverse events following immunization (AEFI).

For peer review only

For peer review only - http://bmjopen.bmj.com/site/about/guidelines.xhtml

INTRODUCTION

Cholera, a preventable and treatable disease is characterized by profuse watery diarrhoea caused by infection of the intestine with the bacterium *Vibrio cholerae.*[1 2] Cholera is a major cause of morbidity and mortality in several countries in sub-Saharan Africa where cholera outbreaks also negatively affect development due to associated high economic burden. [3 4] Between 2010 and 2016 an average 141,918 incident cases annually were reported from sub-Saharan African countries, including Uganda.[5] In Uganda, cholera outbreaks occurred as both endemic and epidemic disease. Epidemic disease occurred in northern and eastern Uganda districts [6] and are thought to be worsened by contamination of safe water due to poor sanitation.[7] Endemic cholera outbreaks occur in districts along the international borders with the Democratic Republic of the Congo (DR Congo), South Sudan and Kenya and along the Great lakes.[6 8] These districts include Hoima, where cholera is endemic. [6 9-11]The World Health Organization (WHO) recommends an integrated approach to cholera prevention where water, sanitation and hygiene (WaSH) interventions are complemented by vaccine campaigns which provide oral cholera vaccine (OCV) to persons living in areas considered high risk.[3] These campaigns may be either preventive, in which the vaccine is targeted to cholera hotspots, or reactive in which the campaign is implemented in response to an outbreak or a humanitarian emergency.[12] There are two OCVs WHO-prequalified currently available from the global stockpile:, Shanchol (Shantha Biotechnics Limited, India), and Euvichol (Eubiologics Co., Ltd., Korea).[3] The standard immunization schedule consists of two doses given at an interval of at least two weeks to all persons in the target area above one year of age. While there is increasing use of OCV to control outbreaks, preventive use is constrained due to inadequate vaccine supply.[13] Since creation of a global OCV stockpile in July, 2013, several OCV campaigns had been successfully implemented[12 13] but it is still important to document national campaign experiences as well as monitoring and evaluation activities, to continually improve the effectiveness and efficiency of vaccine campaigns.

The Ugandan Ministry of Health (MoH) had already prepared plans for OCV campaigns in the areas identified as cholera hotspots starting in the western districts of Uganda (including Hoima), near the border with DR Congo and close to, or adjoining Lake Albert. These hotspot districts and their specified subcounties were confirmed during a national cholera workshop in Kampala on 29-31 January 2018. This workshop led to the development of an application for OCV to the Global Taskforce for Cholera Control (GTFCC) which was submitted on 14 February 2018. The application proposed providing OCV in these identified hotspots as a preventive

For peer review only - http://bmjopen.bmj.com/site/about/guidelines.xhtml

strategy. However, while preparations for these campaigns were underway, an outbreak was declared in Hoima district on 23 February 2018. The earliest cases were identified among DR Congo refugees, but then other cases were seen among the non-refugee Ugandan population. The MoH responded to the outbreak with multisectoral interventions, including proper case management, promotion of access to safe water and improved sanitation (WaSH), enhanced cholera surveillance, as well as infection control and health education. These measures were then supplemented with plans to carry out an emergency OCV campaign. Thus, the plans for a preventive OCV campaign were shifted to an emergency response to control the outbreak. The first doses of vaccine arrived on 28 March and first round of vaccines started on 2 May. The doses for the second round arrived on 29 May and the second found started on 26 June. A door-to-door strategy was used to deliver two doses of vaccine to an estimated 360,000 people, including pregnant women, over the age of one year residing in the four targeted subcounties.

To carry out the campaign, the MoH organized all activities including logistics, community mobilisation and implementation; coordinating ground activities through an assigned point person. Many stakeholders contributed to the campaign including the Hoima district local government, World Health Organisation, UNICEF, UNHCR and Médecins sans Frontiers' (MSF). Prior to the campaign, the stakeholders met in order to define and coordinate their complementary tasks.

This was the first time OCV was deployed in Uganda, and after this initial campaign, the MoH intended to continue with its plans for preventive campaigns in the remaining cholera hotspot districts. Since this was the first OCV use in Uganda, and there was no prior experience to guide responders and implementers, this study was carried out with the aim to document campaign activities and to monitor and evaluate its procedures and outcomes that could guide future OCV campaigns. The issues addressed during this study included the knowledge and practices of the campaign staff, vaccine coverage in the targeted areas, and the knowledge and practices of the community.

MATERIALS AND METHODS

Study setting. Hoima district is located in western Uganda, across Lake Albert from DR Congo. It has a total area of 5735.5 square kilometres and a projected population of 630,000 persons (2018). The district consists of 13 administrative units as follows: 10 sub counties

For peer review only - http://bmjopen.bmj.com/site/about/guidelines.xhtml

(Kyabigambire, Buhimba, Kyangwali, Kabwoya, Bugambe, Kiziranfumbi, Kitoba, Kigorobya, Buseruka and Buhanika), a municipality (Hoima municipality) and two town council (Kigorobya and Buhima town councils). The major economic activities of the population in Hoima are substance agriculture and fishing. Cholera is endemic in the district but the endemicity is localised in some specific subcounties particularly those with fishing communities.[14]

Epidemiologic findings. After the outbreak, an anonymous line list of cases and deaths, by date and stated nationality was used to describe the basic epidemiological features of the outbreak.

Population and design for the Monitoring and Evaluation of the Vaccination Campaign. A cross-sectional study was conducted between May and June 2018 in Hoima districts in the subcounties where the OCV campaign was conducted. This included four sub-counties: Buseruka, Kabwoya, Kangwali, and Kigorobya which together constitute the six administrative units of Kyangwali, Kigorobya, Kabwoya Buseruka, Kigorobya town council and Kyangwali refugee settlement (Old and New) as shown in Figure 1. The study consisted of two sub-studies. Sub-study 1 assessed the knowledge and practices of staff who participated in and contributed to the OCV campaign. Sub-study 2 was a cluster randomised community survey that assessed vaccine coverage, detection of adverse events following immunization (AEFI), and indicators of community knowledge regarding cholera.

Sub-study 1, staff assessment. Staff were assessed on their level of knowledge on the cause of cholera, the importance of safe water in cholera prevention, the target age group for cholera vaccination, and knowledge about AEFI and the procedures for care should subjects experience an AEFI. Staff were surveyed twice with each survey taking place within two weeks after administration of the first and second OCV rounds, respectively. All staff who participated directly by administering the vaccines or indirectly through supervisory roles and who were present at the workstation during the study period were enrolled in the survey. For the staff survey, structured questions were administered on paper questionnaires that allowed for adding text to explain the answers (open-ended questions). There was no list of all workers in the campaign and many of the workers who participated in the second round had left prior to administering the questionnaire; thus, there were fewer respondents in the second round and the proportion of all workers who participated could not be determined precisely.

For peer review only - http://bmjopen.bmj.com/site/about/guidelines.xhtml

Sub-study 2, cluster surveys. Sub-study 2 was carried out with the intention of obtaining information from a representative sample of families in the target area. The study population included each person > one year of age who was living in the OCV targeted area at the time of the vaccination campaign. This sample consisted of 31 household clusters with 4-7 households per cluster. We assumed a household size of five persons. A standard formula for cluster sampling was used to derive the sample size.[15] The sample was increased in order to increase the analytical power and precision of the surveys, using the formula $n = (z^2pq)/d^2$, where 'n' is the number of people desired for the survey, 'd' is the precision of the result, 'z' is the confidence limit, and 'p' and 'q' correspond to the proportion of persons in the population who are immunised and not immunised, respectively. To identify the clusters, a list of villages was obtained from each of the four subcounties targeted for vaccination. From these lists, Excel random number generator (=RANDBETWEENBOTTOM, TOP) was used to select the 31 villages from which households were selected. From each selected village a list of households was obtained from village administrative leader (Local Council (LC) – 1: is the smallest recognised administrative unit in Uganda. It is headed an elected leader called LC-1) and used to identify the household by random selection similar to that used to select the villages or clusters. The subcounty population was obtained from the district planning unit and used to compute the number of households for each sub county as indicated in Table 1.

Table 1. Age and Sex Breakdown of the Participants in the Cluster Survey

Age Group (years)	Male	Female	Total
**1 to 4**	99	93	192
**5 to 14**	236	207	443
**15 to 44**	210	285	495
**45+**	80	65	145
**Total**	625	650	1275

For the household interviews, data were collected through standardized questionnaires during face-to-face interviews conducted by trained research assistants using the local language. Within a selected household the questionnaires were administered to the key respondents

For peer review only

For peer review only - http://bmjopen.bmj.com/site/about/guidelines.xhtml

(head of the HHs), who represented the entire household. If the HH was absent, additional visits were scheduled. In two households, a person could not be located, and the household was dropped. For each vaccinated person, the research assistants assessed cholera immunization status through oral reporting (history) and by reviewing vaccination cards. None of the residents who were approached refused to answer the survey.

Adverse Events Following Immunization (AEFI). The occurrence of AEFIs were assessed by asking for occurrence of symptoms following vaccination. As part of the campaign itself, a routine system for AEFI detection was established in which the vaccine team members advised vaccinees to report to a health worker or to seek care at a health facility if they experienced symptoms following immunization. By contrast, the AEFI surveillance in this study asked the participants who participated in the cluster survey about symptoms they may have experienced. This AEFI sub-study was thus, designed to enhance our understanding of potential AEFIs which may not have been reported through the routine AEFI surveillance.

AEFIs were categorised for each individual member of the household who received a dose of OCV as follows. They were considered mild if the symptoms did not interfere with normal activities; moderate if they interfered somewhat; and severe if the symptoms prevented the individual from continuing normal activities. Persons who reported to be having ongoing symptoms > 72 hours were advised to visit the nearest health facility for more care.

Data collection and analysis. Data from the community surveys were collected by tablet computers using *Kobo Collect* , https://www.kobotoolbox.org/ installed to record the responses in the field. Data were cleaned, coded and stored in Stata Version 14. Data were analysed to generate frequencies, percentages or proportions and means. Comparisons between groups, such as sub-counties or age strata was done using Chi Square or 2 x X tables with the calculation of odds ratios and 95% confidence intervals. Multivariate analysis was done using conditional logistic regression, or Poisson regression for analysis of uncommon events. Continuous variables were analysed and compared using Student's T test or analysis of variance (ANOVA). The results of analysis were presented in the form of graphs, tables, charts and means and were included in interim and end of campaign reports.

Quality Assurance. Research assistants were trained on data collection methods and were able to consult field supervisors and the principal investigator on any issue not clear to them. For

For peer review only - http://bmjopen.bmj.com/site/about/guidelines.xhtml

quality assurance, the survey supervisors revisited about 10% of the households, not to collect the data again, but to ensure that they were not skipped by the interviewers for eligible respondents. The surveys were conducted within two weeks of completion of the second round of the vaccination campaign to minimize recall bias.

Ethical Considerations. This study was conducted as part of the routine MoH operational research for improvement of health services; however ethical issues were considered and addressed. The proposal was approved by the Makerere University School of Public Health Institutional Review Board (MaKSPH IRB) and Uganda National Council of Science and Technology. Written informed consent was obtained from all participants, including for both the staff and community surveys. Participation in the study was voluntary and respondents were free to opt out at any stage of the interviews.

Confidentiality was observed at all stages of the study. No names or personal identifiers were included on the questionnaires. The research assistants underwent training on interview techniques, neutrality and research ethics. The benefits of the study to the staff included the ability to express themselves, provide feedback and observations that in turn might lead to improvements in supportive services for their training and work.

Patient and Public Involvement. This research was done without research subject involvement. There was not enough time to involve the subjects prior to the vaccine campaign. They were also not invited to contribute to the writing or editing of this document for readability or accuracy. However, the findings of the study were disseminated to the Hoima district administration, MoH and other policymakers to use them to strengthen health service interventions and future OCV campaigns.

RESULTS

***The epidemic curve*.** As shown on Figure 2, the epidemic was declared on the 8th week of 2018. A total of 2,122 cases with 44 deaths (case fatality rate, 2.1%) was reported during the outbreak. Sixty seven percent (1,410) and 64% (28) of the deaths occurred during the first two weeks of the outbreak. Many of the cases and deaths (1276 and 32, or 60% and 73%, respectively) occurred among persons who were from DR Congo, nearly all refugees who developed cholera symptoms soon after arrival in Uganda. Among the 44 deaths reported, 25 (57%) occurred in the community, not in the health facility. Nineteen of the fatal cases were treated at the health facility; the CFR for facility-treated patients was 0.9%. The vaccination

For peer review only - http://bmjopen.bmj.com/site/about/guidelines.xhtml

campaign, originally planned as a preventive OCV campaign, was changed to an emergency response intended to control the outbreak. Because the outbreak was so sudden and was short-lived, the campaign could only be initiated after the outbreak had already declined.

Sub-study I, staff survey. A total of 242 and 125 staff were interviewed in the first and second knowledge and practice (KP) surveys (KP1 and KP2). Most respondents were vaccination team members (89% and 87% in rounds 1 and 2, respectively). Almost all the respondents were knowledgeable about the cause of cholera, the importance of safe water in cholera prevention and the vaccine target group, but were less knowledgeable regarding potential adverse events following administration (AEFI) or how to advise vaccinees with 29% and 16% being less informed about AEFI during the first and second surveys.

When staff were asked to suggest areas that needed improvement in future OCV campaigns, more than 10% suggested more timely payment of allowances, more time to sensitize and inform the communities on the benefits of the vaccine, and better transportation and facilitation allowances (payments to health workers to cover the cost they incurred when administering the vaccines or conducting activities related to the OCV campaign). Other suggestions included use of both static and mobile vaccination points, provision of gumboots, umbrellas, and more areas for vaccine storage in subcounties where vaccine would be more accessible, more workers for hard to reach areas, and an increase in the number of vaccine days.

Sub-study II, community survey results. The community surveys were carried out in four sub-counties of Buseruka, Kabwoya, Kyangwali and Kigorobya as shown in Figure 1 and Table 1. A total of 209 households, including 1,259 individuals, were surveyed. Most (96%) of the respondents were household heads or their spouses. All respondents confirmed that they were living in the targeted OCV area at the time of the campaign. 51% of the respondents had primary education, 17% had secondary education, 1% had tertiary education, and the remaining 31% had no education. The respondents were aged 18 – 89 years with a mean age of 40 years. Both sexes were present, with no statistically significant difference.

By verbal reports, 93% of the residents received at least one dose and 78% received two doses of OCV. Based on the vaccination card to confirm the vaccination status, the two-dose coverage was 62% (95%CI: 59.4-64.9). 91% of residents received vaccine during the first round and 80% received vaccine during the second round. Coverage rates are shown on Table 2.

For peer review only

Table 2: Vaccination coverage post OCV campaign, Hoima District, Uganda, 2018.

	Round 1	Round 2	Received only one dose	Received two doses	Received at least one dose	Total surveyed
**Reported number (%)**	1,164 (91.2)	1,027 (80.5)	195 (15.3)	998 (78.3)	1193 (93.6)	1275
**95% CI**	89.7-928	78.3-82.7	13.4-17.4	76.0-80.6	92.2-94.9	
**Confirmed by availability of the vaccination card (%)**	1,065 (83.5)	823 (64.5)	304 (23.8)	792 (62.1)	1096 (86.0)	
**95% CI**	81.5-85.6	62.0-67.2	21.6-26.3	59.5-64.8	84.1-87.9	

Among those who did not receive a dose of vaccine, over half of these missed doses (255 missed doses during the two rounds) were because the person was not at home at the time of vaccination or was out of town. In a few cases, the vaccine team missed the household, accounting for 53 missed doses. Refusing to take vaccine was not reported.

Reported AEFIs. Overall, 71 individuals of 1,259 respondents (5.6%) reported an AEFI (Table 3).

Table 3: Treatment and resolution of Adverse Events Following Immunization in Hoima District, Uganda, 2018.

Symptoms	Treatment		Status		
	No treatment (%)	Treated (%)	Recovered (%)	Ongoing (%)	Improved, not to baseline (%)
**Mild**	24(80.0)	6(20.0)	29(96.7)	1(3.3)	0(0.0)
**Moderate**	11(39.3)	17(60.7)	23(82.1)	3(10.7)	2(7.1)

For peer review only

**Severe**	8(61.5)	5(38.5)	10(76.9)	0(0.0)	3(23.1)

Most of these were considered mild or moderate, but 8 (0.6%) persons reported an AEFI as severe. Most (60%) of the persons reporting an AEFI did not seek treatment including 60% of those reporting a severe AEFI. 29.6% of the reported adverse events occurred in the first round, 40.9% in the second round and 29.6% in both rounds. The most common symptoms were abdominal pain (15), diarrhoea, (9), fever, nausea and headache (each 6) reports. Table 4 provides additional information on the AEFIs.

Table 4. Onset and frequency of symptoms reported as adverse events

	<6 hours	6-12 hours	12-24 hours	1-2 days	3-7 days	8-14 days	Total
Diarrhoea	3	2	2	1	1	0	9
Vomiting	3	0	0	0	2	0	5
Nausea	6	0	0	0	0	0	6
Abdominal pain	15	0	0	0	0	0	15
Stomach gurgling	0	3	0	1	0	0	4
Mouth ulcers	0	0	0	0	0	1	1
cough	1	1	0	2	1	1	6
Felt feverish	1	1	0	0	6	1	9
Poor appetite	1	0	0	0	0	0	1
Dizziness	0	3	0	0	0	0	3
Fainted	0	1	0	0	0	0	1
Itching	0	0	0	0	0	1	1
Weakness	0	0	0	0	1	0	1
Headache	3	0	2	1	0	0	6
Other	1	0	0	2	0	0	3
Total	34	11	4	7	11	4	71

For peer review only

For peer review only - http://bmjopen.bmj.com/site/about/guidelines.xhtml

Community knowledge. Most respondents knew the major signs and symptoms of cholera, that it was related to contaminated water or food, the importance of drinking safe water and hand washing. Following the campaign, they also understood that vaccine was also a way to prevent cholera. There was a statistically significant association between education level and knowledge about OCV, with those having at least a primary school education being two and a half times as likely to know the number of required doses as compared to those with no education (RO 2.46, 95% CI 1.09, 5.59 [P = 0.023].

DISCUSSION

Our findings suggest that the OCV campaign in Hoima successfully provided vaccine to a very large proportion of the target population in these four subcounties in western Uganda. 93% reported receiving at least one dose and 78% reported receiving two doses among residents. Given the mobile and transient nature of this population, this was noteworthy, and suggests that even better coverage may be possible for more settled populations in Uganda. Refusal to take vaccine was not a constraint to the campaign, but not finding people at home did result in many missed vaccinations. High coverage is especially important when one is attempting to achieve herd protection. Since it is estimated that herd protection can be achieved with a coverage even lower than 90%;[16] the high coverage achieved in this campaign would be expected to induce significant protection among even those who did not receive vaccine.

It was noted in the administrative report from the MoH and during a stakeholder's meeting that one of the reasons for the reduction in the coverage during the second OCV dose was the unpredictable campaign dates for the second round. The vaccine for the second round had to be shipped and cleared through customs, and the timing for this was not certain. To avoid this problem in the future, a mechanism needs to be established to provide a better timeline for receipt of the vaccine shipments.

Inclusion of staff KP survey contributed to the success by identifying gaps amongst the staff knowledge and performance. After the first staff KP survey, these gaps were communicated to the MoH and the stakeholders responded with appropriate actions to ensure that these gaps were addressed prior to the second round.

As with previous OCV campaigns very few AEFI were reported.[17 18] Most of adverse events were considered mild or moderate and were self-limited. Despite the low prevalence of AEFI,

For peer review only - http://bmjopen.bmj.com/site/about/guidelines.xhtml

the survey exposed the need to better inform the community about seeking treatment for more severe adverse events or for those that do not quickly resolve. This was especially true for those for families with little education who were less likely to seek medical attention for severe AEFI (data not shown).

Most other OCV campaigns have also reported high coverage rates. These have included reports from Bangladesh[19 20], Malawi[21 22], Mozambique[17],Democratic Republic of Congo [23], Zambia [24], South Sudan [25 26], Iraq[27], Haiti[28], and Guinea [29]. Clearly, OCV is well accepted among these very diverse population groups where the vaccine campaigns have been carried out.

Important limitations of this study included only one series of surveys for the community, rather than one following each vaccination round. Learning from the community prior to the campaign as well as immediately after the first dose might have provided feedback to the teams that would have improved the coverage for the second round and would have further increased two dose coverage rates. Similarly, more direct observation of the training and coordination meetings would have been useful to independently assess the efficiency and effectiveness of these training and coordination meetings.

The community KP survey did not include questions on attitudes regarding cholera; it was not a KAP survey. Since the survey had to be carried out very quickly following the campaign, and since the survey was targeted to identify issues that would be immediately relevant to campaign performance, it was felt that understanding attitudes regarding cholera, even though important, would have required other qualitative methods requiring more time than was available.

In this outbreak 2,122 cholera cases and 44 deaths were reported, nearly all before the OCV campaign and over half occurred in the first two weeks of the outbreak. Of note, the outbreak started in February 2018 at about the same time the application for preventive use of OCV was being was submitted. The original application proposed a series of preventive campaigns over the next year, and Hoima, as well as neighbouring districts in western Uganda, were targeted for vaccination in the first round of these preventive campaigns. However, when the outbreak was identified, plans were quickly shifted so that an emergency campaign could be implemented to control the outbreak. Even though this emergency response was planned as

For peer review only - http://bmjopen.bmj.com/site/about/guidelines.xhtml

quickly as possible, in fact, the outbreak was nearly over before the vaccine campaign could start.

Furthermore, though the outbreak started with the influx of the refugees from DRC into Uganda, it quickly spread to the refugee host communities in Hoima. Therefore, to prevent rapid spread, improvement of cholera prevention measures for both the refugees and the host communities is paramount during resettlement.

CONCLUSION

This study suggests that the integrated OCV campaign in Hoima district to prevent cholera was successful and achieved a high level of coverage in this population that was targeted to be at risk. However, there was a need to devote more effort on community sensitisation on the benefits of vaccination, as well as improving some logistic support during the campaign.

While a rapid response to this outbreak was appropriate, in fact, even with a rapidly organized campaign, the outbreak was largely over before the vaccine could be given; thus, the vaccine likely had little impact on this outbreak. Nevertheless, this area had already been identified as a hotspot, and it would have been targeted if the planned preventive campaign had proceeded as originally planned. Planners must realize that an area identified as a hotspot might experience an outbreak while preparations are underway for a preventive campaign and take this into account when allocating vaccine for preventive vs emergency campaigns. Since these are areas where cholera risk is high, outbreaks are likely to occur in these areas if there are delays in implementing preventive campaigns.

LEGENDS

Figure 1: Map of Hoima District, showing sub-counties that received OCV and households where interviews were conducted (red dots)

For peer review only - http://bmjopen.bmj.com/site/about/guidelines.xhtml

Figure 2. Epicurve of the Hoima Outbreak, 2018 with events identified in response to the outbreak

Acknowledgements

The authors thank the persons and institutions who supported Hoima district OCV monitoring and evaluation study. Special thanks are extended to the research assistants who dedicated their time to meticulously collect the data and the persons who collaborated in the preparation of the different key documents used in this study. We thank the staff, the communities in the study area and the local leadership of Hoima districts for the cooperation during this study. We are grateful to Ambrose Buyinza Wabwire for technical support with drawing of the maps.

We thank the Ministry of Health leadership for guidance; the Office of the Prime Minister (OPM) for the permission to access data on the refugee population. We are grateful to the following institutions and bodies; Ministry of Health, Makerere University School of Public Health, World Health Organisation, UNICEF and UNHCR for technical guidance.

Footnotes

Contributors: Conceived and designed the study: GB, MR, WAB, ABS, CGO, DAS. Discussed, critically revised and approved the study protocol: GB, MR, WAB, CGO, DAS. Performed the research: GB, MR, WAB, AO, FR, IA. Analysed the data: GB, MR, WAB, AO, DAS. Wrote the first draft: GB, MR. Wrote the final DAS. Elaborated, discussed and approved the final version: GB, MR, AB, ABS, AO, FR, IA, CGO, and DAS.

Funding: Funding for the study was provided by the Bill and Melinda Gates Foundation (OPP1148763) which provided financial support through the John Hopkins University, Delivery of Oral Cholera Vaccine Effectively (DOVE) project. The funding agency had no role in collecting, analysing or interpreting the results.

Declaration of the conflict of interest

The authors have no conflict of interest to declare.

For peer review only - http://bmjopen.bmj.com/site/about/guidelines.xhtml

Additional data sharing: No additional data available.

For peer review only

For peer review only - http://bmjopen.bmj.com/site/about/guidelines.xhtml

REFERENCES

1. Clemens JD, Nair GB, Ahmed T, et al. Cholera. *Lancet* 2017 doi: 10.1016/S0140-6736(17)30559-7
2. Butler T. New developments in the understanding of cholera. *Current gastroenterology reports* 2001;3(4):315-21. [published Online First: 2001/07/27]
3. Cholera vaccines: WHO position paper - August 2017. *Wkly Epidemiol Rec* 2017;92(34):477-98.
4. Kirigia JM, Sambo LG, Yokouide A, et al. Economic burden of cholera in the WHO African region. *BMC Int Health Hum Rights* 2009;9:8. doi: 10.1186/1472-698X-9-8
5. Lessler J, Moore SM, Luquero FJ, et al. Mapping the burden of cholera in sub-Saharan Africa and implications for control: an analysis of data across geographical scales. *Lancet* 2018;391(10133):1908-15. doi: 10.1016/S0140-6736(17)33050-7
6. Bwire G, Ali M, Sack DA, et al. Identifying cholera "hotspots" in Uganda: An analysis of cholera surveillance data from 2011 to 2016. *PLoS Negl Trop Dis* 2017;11(12):e0006118. doi: 10.1371/journal.pntd.0006118
7. Cummings MJ, Wamala JF, Eyura M, et al. A cholera outbreak among semi-nomadic pastoralists in northeastern Uganda: epidemiology and interventions. *Epidemiology and infection* 2012;140(8):1376-85. doi: 10.1017/S0950268811001956 [published Online First: 2011/09/29]
8. Bwire G, Mwesawina M, Baluku Y, et al. Cross-Border Cholera Outbreaks in Sub-Saharan Africa, the Mystery behind the Silent Illness: What Needs to Be Done? *PLoS One* 2016;11(6):e0156674. doi: 10.1371/journal.pone.0156674
9. Bwire G, Malimbo M, Maskery B, et al. The burden of cholera in Uganda. *PLoS Negl Trop Dis* 2013;7(12):e2545. doi: 10.1371/journal.pntd.0002545
10. Oguttu DW, Okullo A, Bwire G, et al. Cholera outbreak caused by drinking lake water contaminated with human faeces in Kaiso Village, Hoima District, Western Uganda, October 2015. *Infect Dis Poverty* 2017;6(1):146. doi: 10.1186/s40249-017-0359-2
11. Alajo SO, Nakavuma J, Erume J. Cholera in endemic districts in Uganda during El Nino rains: 2002-2003. *Afr Health Sci* 2006;6(2):93-7. doi: 10.5555/afhs.2006.6.2.93 [published Online First: 2006/08/19]
12. Deployments from the oral cholera vaccine stockpile, 2013-2017. *Wkly Epidemiol Rec* 2017;92(32):437-42.
13. Odevall L, Hong D, Digilio L, et al. The Euvichol story - Development and licensure of a safe, effective and affordable oral cholera vaccine through global public private partnerships. *Vaccine* 2018;36(45):6606-14. doi: 10.1016/j.vaccine.2018.09.026
14. Bwire G, Munier A, Ouedraogo I, et al. Epidemiology of cholera outbreaks and socio-economic characteristics of the communities in the fishing villages of Uganda: 2011-2015. *PLoS Negl Trop Dis* 2017;11(3):e0005407. doi: 10.1371/journal.pntd.0005407
15. Henderson RH, Sundaresan T. Cluster sampling to assess immunization coverage: a review of experience with a simplified sampling method. *Bull World Health Organ* 1982;60(2):253-60.

For peer review only - http://bmjopen.bmj.com/site/about/guidelines.xhtml

16. Longini IM, Jr., Nizam A, Ali M, et al. Controlling endemic cholera with oral vaccines. *PLoS medicine* 2007;4(11):e336. doi: 10.1371/journal.pmed.0040336 [published Online First: 2007/11/30]

17. Sema Baltazar C, Rafael F, Langa JPM, et al. Oral cholera vaccine coverage during a preventive door-to-door mass vaccination campaign in Nampula, Mozambique. *PLoS One* 2018;13(10):e0198592. doi: 10.1371/journal.pone.0198592

18. Phares CR, Date K, Travers P, et al. Mass vaccination with a two-dose oral cholera vaccine in a long-standing refugee camp, Thailand. *Vaccine* 2016;34(1):128-33. doi: 10.1016/j.vaccine.2015.10.112

19. Khan AI, Khan IA, Siddique SA, et al. Feasibility, coverage and cost of oral cholera vaccination conducted by icddr,b using the existing national immunization service delivery mechanism in rural setting Keraniganj, Bangladesh. *Hum Vaccin Immunother* 2019;15(6):1302-09. doi: 10.1080/21645515.2018.1528833

20. Uddin MJ, Wahed T, Saha NC, et al. Coverage and acceptability of cholera vaccine among high-risk population of urban Dhaka, Bangladesh. *Vaccine* 2014;32(43):5690-5. doi: 10.1016/j.vaccine.2014.08.021

21. Grandesso F, Rafael F, Chipeta S, et al. Oral cholera vaccination in hard-to-reach communities, Lake Chilwa, Malawi. *Bull World Health Organ* 2018;96(12):817-25. doi: 10.2471/BLT.17.206417

22. Sauvageot D, Saussier C, Gobeze A, et al. Oral cholera vaccine coverage in hard-to-reach fishermen communities after two mass Campaigns, Malawi, 2016. *Vaccine* 2017;35(38):5194-200. doi: 10.1016/j.vaccine.2017.07.104

23. Massing LA, Aboubakar S, Blake A, et al. Highly targeted cholera vaccination campaigns in urban setting are feasible: The experience in Kalemie, Democratic Republic of Congo. *PLoS Negl Trop Dis* 2018;12(5):e0006369. doi: 10.1371/journal.pntd.0006369

24. Poncin M, Zulu G, Voute C, et al. Implementation research: reactive mass vaccination with single-dose oral cholera vaccine, Zambia. *Bull World Health Organ* 2018;96(2):86-93. doi: 10.2471/BLT.16.189241

25. Parker LA, Rumunu J, Jamet C, et al. Neighborhood-targeted and case-triggered use of a single dose of oral cholera vaccine in an urban setting: Feasibility and vaccine coverage. *PLoS Negl Trop Dis* 2017;11(6):e0005652. doi: 10.1371/journal.pntd.0005652

26. Porta MI, Lenglet A, de Weerdt S, et al. Feasibility of a preventive mass vaccination campaign with two doses of oral cholera vaccine during a humanitarian emergency in South Sudan. *Trans R Soc Trop Med Hyg* 2014;108(12):810-5. doi: 10.1093/trstmh/tru153

27. Lam E, Al-Tamimi W, Russell SP, et al. Oral Cholera Vaccine Coverage during an Outbreak and Humanitarian Crisis, Iraq, 2015. *Emerg Infect Dis* 2017;23(1):38-45. doi: 10.3201/eid2301.160881

28. Tohme RA, Francois J, Wannemuehler K, et al. Oral Cholera Vaccine Coverage, Barriers to Vaccination, and Adverse Events following Vaccination, Haiti, 2013. *Emerg Infect Dis* 2015;21(6):984-91. doi: 10.3201/eid2106.141797

29. Luquero FJ, Grout L, Ciglenecki I, et al. First outbreak response using an oral cholera vaccine in Africa: vaccine coverage, acceptability and surveillance of adverse events, Guinea, 2012. *PLoS Negl Trop Dis* 2013;7(10):e2465. doi: 10.1371/journal.pntd.0002465

For peer review only - http://bmjopen.bmj.com/site/about/guidelines.xhtml

528

For peer review only - http://bmjopen.bmj.com/site/about/guidelines.xhtml

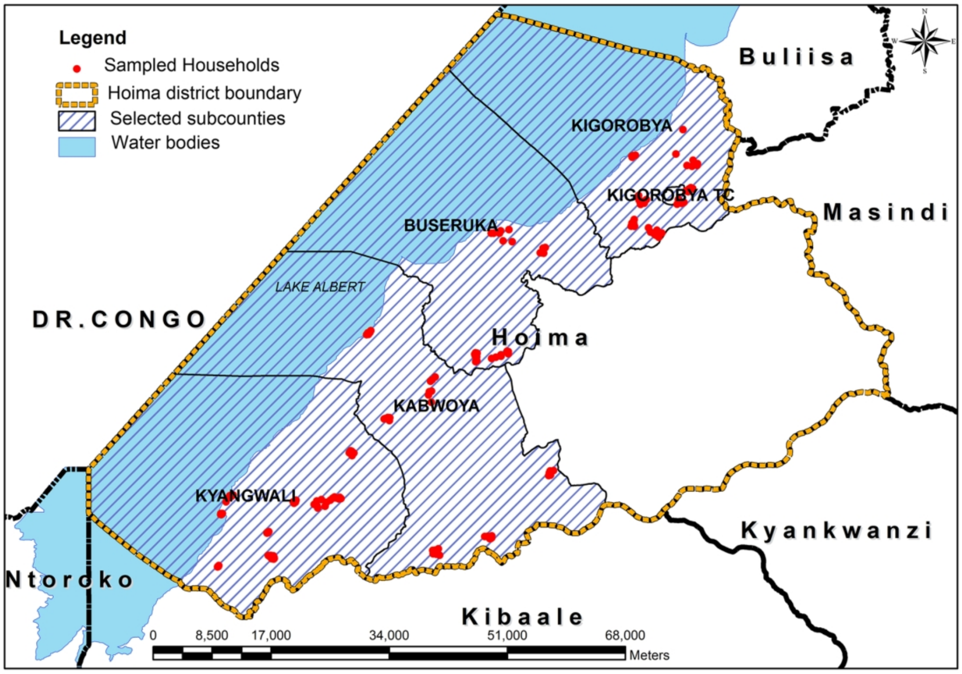

170x119mm (300 x 300 DPI)

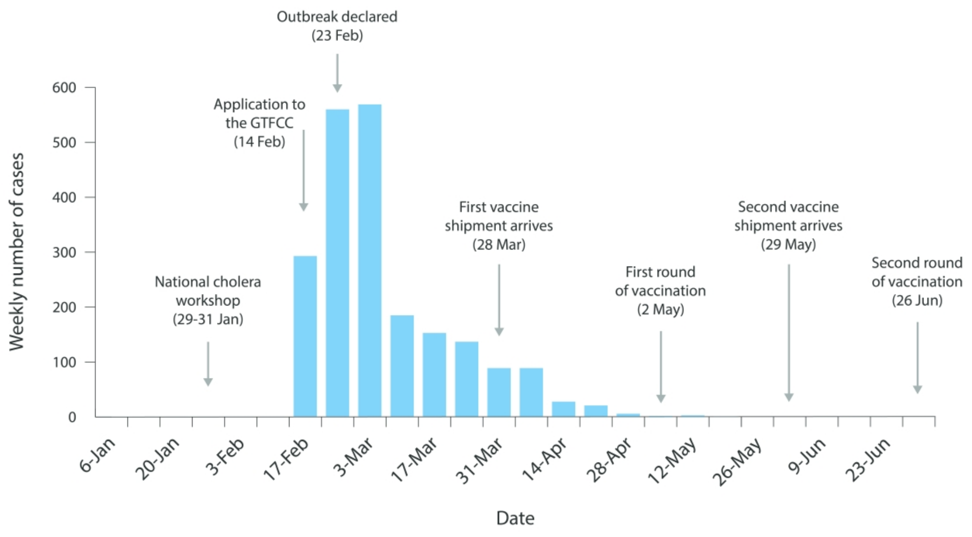

542x305mm (300 x 300 DPI)

BMJ Open

Evaluation of an integrated oral cholera vaccine campaign in response to a cholera outbreak in Hoima District, Uganda

Journal:	*BMJ Open*
Manuscript ID	bmjopen-2020-038464.R1
Article Type:	Original research
Date Submitted by the Author:	05-Oct-2020
Complete List of Authors:	Bwire, Godfrey; Republic of Uganda Ministry of Health, Community Health<br>Roskosky, Mellisa; Johns Hopkins University Bloomberg School of Public Health, International Health<br>Ballard, Anne; Johns Hopkins University Bloomberg School of Public Health, Center for Communications Programs<br>Brooks, W; Johns Hopkins University Bloomberg School of Public Health, International Health<br>Alfred, Okello; Makerere University College of Health Sciences<br>Rafael, Florentina; World Health Organization, Infectious Hazard Management<br>Ampeire, Immaculate; Republic of Uganda Ministry of Health, Expanded Programme on Immunization<br>Orach, Christopher; Makerere University College of Health Sciences<br>Sack, David; Johns Hopkins University Bloomberg School of Public Health, International Health
**Primary Subject Heading**:	Global health
Secondary Subject Heading:	Epidemiology, Health services research, Infectious diseases, Public health
Keywords:	Gastrointestinal infections < GASTROENTEROLOGY, International health services < HEALTH SERVICES ADMINISTRATION & MANAGEMENT, Epidemiology < INFECTIOUS DISEASES, Infection control < INFECTIOUS DISEASES, Public health < INFECTIOUS DISEASES, Tropical medicine < INFECTIOUS DISEASES

SCHOLARONE™
Manuscripts

I, the Submitting Author has the right to grant and does grant on behalf of all authors of the Work (as defined in the below author licence), an exclusive licence and/or a non-exclusive licence for contributions from authors who are: i) UK Crown employees; ii) where BMJ has agreed a CC-BY licence shall apply, and/or iii) in accordance with the terms applicable for US Federal Government officers or employees acting as part of their official duties; on a worldwide, perpetual, irrevocable, royalty-free basis to BMJ Publishing Group Ltd ("BMJ") its licensees and where the relevant Journal is co-owned by BMJ to the co-owners of the Journal, to publish the Work in this journal and any other BMJ products and to exploit all rights, as set out in our licence.

The Submitting Author accepts and understands that any supply made under these terms is made by BMJ to the Submitting Author unless you are acting as an employee on behalf of your employer or a postgraduate student of an affiliated institution which is paying any applicable article publishing charge ("APC") for Open Access articles. Where the Submitting Author wishes to make the Work available on an Open Access basis (and intends to pay the relevant APC), the terms of reuse of such Open Access shall be governed by a Creative Commons licence – details of these licences and which Creative Commons licence will apply to this Work are set out in our licence referred to above.

Other than as permitted in any relevant BMJ Author's Self Archiving Policies, I confirm this Work has not been accepted for publication elsewhere, is not being considered for publication elsewhere and does not duplicate material already published. I confirm all authors consent to publication of this Work and authorise the granting of this licence.

For peer review only

Evaluation of an integrated oral cholera vaccine campaign in response to a cholera outbreak in Hoima District, Uganda

Godfrey Bwire[1], Mellisa Roskosky[2], Anne Ballard Sara[2], W. Abdullah Brooks[2], Alfred Okello[3], Florentina Rafael [4], Immaculate Ampeire[5], Christopher Garimoi Orach[3], David A. Sack[2].

Authors' affiliations:

1. Ministry of Health, Department of Community Health, Kampala Uganda.
2. Johns Hopkins Bloomberg School of Public Health, Department of International Health, Baltimore, Maryland USA.
3. Makerere University, College of Health Sciences, Kampala, Uganda.
4. World Health Organization, Department of Infectious Hazard Management, Geneva, Switzerland.
5. Ministry of Health, Uganda National Expanded Program on Immunization

*Corresponding author.

Professor David A. Sack, M, D.
Johns Hopkins Bloomberg School of Public Health
615 North Wolfe Street
Baltimore, Maryland 21205 USA
Phone +1 443 287 8795
Email: dsack1@jhu.edu

Number of words (Excluding abstract): 4,586
Number of words in abstract: 258
Number of tables: 4
Number of figures: 2
Number of references: 33
Number of supplemental materials: 0

Key words: cholera, vaccination, Uganda, monitoring, coverage, outbreak, sub-Saharan Africa, oral cholera vaccine, Vaccine campaign

For peer review only

For peer review only - http://bmjopen.bmj.com/site/about/guidelines.xhtml

ABSTRACT

Objectives To evaluate the quality and coverage of the campaign to distribute oral cholera vaccine during a cholera outbreak in Hoima, Uganda to guide future campaigns of cholera vaccine.

Design Survey of communities targeted for vaccination to determine vaccine coverage rates and perceptions of the vaccination campaign, and a separate survey of vaccine staff who carried out the campaign.

Setting Hoima District, Uganda.

Participants Representative clusters of households residing in the communities targeted for vaccination and staff members who conducted the vaccine campaign.

Results Among 209 households (1,274 individuals) included in the coverage survey, 1193 (94%) reported receiving at least one OCV dose and 998 (78%) reported receiving two doses. Among vaccinated individuals, minor complaints were reported by 71 persons (5.6%). Individuals with 'some' education (primary school or above) were more knowledgeable regarding the required OCV doses compared to non-educated (p value = 0.03). Factors negatively associated with campaign implementation included community sensitisation time, staff payment, and problems with field transport. Although the campaign was carried out quickly, the outbreak was over before the campaign started. Most staff involved in the campaign (93%) were knowledgeable about cholera control; however, 29% did not clearly understand how to detect and manage adverse events following immunization.

Conclusion The campaign achieved high OCV coverage, but the surveys provided insights for improvement. To achieve high vaccine coverage, more effort is needed for community sensitisation, and additional resources for staff transportation and timely payment for campaign staff is required. Pre and post-test assessment of staff training can identify and address knowledge and skill gaps.

For peer review only - http://bmjopen.bmj.com/site/about/guidelines.xhtml

Strengths and limitations of this study

- The cluster survey of households in communities targeted for vaccination efficiently documented actual coverage in the target population.
- The household surveys were able to identify constraints to the campaign, especially in less educated subgroups
- The household surveys identified mild adverse events not detected by routine AEFI surveillance (Adverse Events Following Immunization) during the campaign. Understanding these perceived AEFIs should be recognized to ensure good communications between communities and health officials.
- Evaluation of the vaccination staff immediately following each round identified ways to improve performance in the next round.
- Monitoring and evaluation of the oral cholera vaccination campaign in Hoima district was useful to guide future cholera prevention activities since the campaign was the first of its kind. in Uganda.

For peer review only

INTRODUCTION

Cholera, a preventable and treatable disease is characterized by profuse watery diarrhoea caused by infection of the intestine with the bacterium *Vibrio cholerae.*[1] Cholera is a major cause of morbidity and mortality in several countries in sub-Saharan Africa where cholera outbreaks also negatively affect development due to associated high economic burden. [2 3] Between 2010 and 2016 an average 141,918 incident cases annually were reported from sub-Saharan African countries, including Uganda.[4] In Uganda, cholera outbreaks occurred as both endemic and epidemic disease. Epidemic disease occurred in northern and eastern Uganda districts [5] and are thought to be worsened by contamination of water due to poor sanitation.[6] Cholera outbreaks especially occur in districts along the international borders with the Democratic Republic of the Congo (DR Congo), South Sudan and Kenya and along the Great Lakes.[5 7] These districts include Hoima, where cholera is endemic. [5 8-10]

There has been debate in the public health community on best practices for endemic and epidemic cholera disease control, with some preferring to focus on WASH interventions, and others advocating for OCV for both endemic and epidemic disease control.[11]. In part, this has been facilitated by a relative lack of experience with OCV and concern that excess reliance on vaccine might negatively affect essential infrastructural development and hygienic practices. The World Health Organization (WHO) recommends an integrated approach to cholera prevention where water, sanitation and hygiene (WaSH) interventions are complemented by vaccine campaigns which provide oral cholera vaccine (OCV) to persons living in areas considered high risk.[2 12] These vaccine campaigns may be either preventive, in which the vaccine is targeted to cholera hotspots, or reactive in which the campaign is implemented in response to an outbreak or a humanitarian emergency.[13]

Two WHO-prequalified currently OCVs are available from the global stockpile: Shanchol (Shantha Biotechnics Limited, India) and Euvichol (Eubiologics Co., Ltd., Korea).[2] The standard immunization schedule consists of two doses given at an interval of at least two weeks to all persons in the target area above one year of age. While there is increasing use of OCV to control outbreaks, preventive use is constrained due to inadequate vaccine supply.[14] Since creation of a global OCV stockpile in July, 2013, several OCV campaigns had been successfully implemented[13 14] but it is still important to document national campaign experiences as well as monitoring and evaluation activities, to continually improve the effectiveness and efficiency of vaccine campaigns.

For peer review only - http://bmjopen.bmj.com/site/about/guidelines.xhtml

The Ugandan Ministry of Health (MoH) had prepared plans for OCV campaigns in the areas identified as cholera hotspots starting in the western districts of Uganda (including Hoima), near the border with DR Congo and close to, or adjoining Lake Albert. These hotspot districts and their specified sub-counties were confirmed during a national cholera workshop in Kampala on 29-31 January 2018. This workshop led to the development of an application for OCV to the Global Taskforce for Cholera Control (GTFCC) which was submitted on 14 February 2018. The application proposed providing OCV in these identified hotspots as a preventive strategy. However, while preparations for these campaigns were underway, an outbreak was declared in Hoima district on 23 February 2018. The earliest cases were identified among DR Congo refugees, but then other cases were seen among the non-refugee Ugandan population. The MoH responded to the outbreak with multisectoral interventions, including proper case management, promotion of access to safe water and improved sanitation (WaSH), enhanced cholera surveillance, as well as infection control and health education. These measures were then supplemented with plans for an emergency OCV campaign. Thus, the original plans for a preventive OCV campaign were shifted to an emergency response to control the outbreak. The first doses of vaccine arrived on 28 March and the first round of vaccinations started on 2 May. The doses for the second round arrived on 29 May and the second round started on 26 June. A door-to-door strategy was used to deliver two doses of vaccine to an estimated 360,000 people, including pregnant women, over the age of one year residing in the four targeted sub-counties. To carry out the campaign, the MoH organized all activities including logistics, community mobilisation and implementation, coordinating ground activities through an assigned point person. Many stakeholders contributed to the campaign including the Hoima district local government, WHO, UNICEF, UNHCR and Médecins sans Frontiers (MSF). Prior to the campaign, the stakeholders met to define and coordinate their complementary tasks.

The epidemic curve based on a line list of cases and deaths by date and stated nationality is shown on Figure 1. Over the course of the outbreak, 2,122 cases with 44 deaths (case fatality rate, 2.1%) were reported. Sixty six percent (1,410) of the cases and 64% (28) of the deaths occurred during the first two weeks of the outbreak. Many of the cases and deaths (1276 and 32, or 60% and 73%, respectively) occurred among persons who were from DR Congo, and the refugees developed cholera symptoms soon after arrival in Uganda. Among the 44 deaths reported, 25 (57%) occurred in the community, not in the health facility. Nineteen of the fatal cases were treated at the health facility; the case fatality ratio (CFR) for facility-treated patients was 0.9%. Although the emergency vaccination campaign intended to control the outbreak,

For peer review only - http://bmjopen.bmj.com/site/about/guidelines.xhtml

because the outbreak was so sudden and so short-lived, the campaign could only be initiated after the outbreak had already declined.

Rationale. While vaccines are commonly used in Uganda, especially through the longstanding EPI program (Expanded Programme on Immunization), this was the first OCV use in Uganda, and there was no prior experience to guide responders and implementers. Thus, this study was carried out with the aim to document campaign activities and to monitor and evaluate its procedures and outcomes that could guide future OCV campaigns. The issues addressed during this study included the knowledge and practices of the campaign staff, vaccine coverage in the targeted areas, and the knowledge and practices of the community. After this initial campaign, the MoH continued its plans for preventive campaigns in the remaining cholera hotspot districts, informed by the lessons learned from this initial emergency use campaign. In an effort to document the impact of OCV on an outbreak in a setting with endemic disease, we undertook this monitoring and evaluation exercise, as described below.

MATERIALS AND METHODS

Study setting. Hoima district is located in western Uganda, across Lake Albert from DR Congo. It has a total area of 5735.5 square kilometres and a projected population of 630,000 persons (2018). The district consists of 13 administrative units as follows: 10 sub-counties (Kyabigambire, Buhimba, Kyangwali, Kabwoya, Bugambe, Kiziranfumbi, Kitoba, Kigorobya, Buseruka and Buhanika), a municipality (Hoima municipality) and two town councils (Kigorobya and Buhima town councils). The major economic activities of the population in Hoima are subsistence agriculture and fishing. Cholera is endemic in the district but the endemicity is localised in some specific sub-counties particularly those with fishing communities.[15] The sub-counties targeted for OCV included Buseruka, Kabwoya, Kangwali, and Kigorobya which together constitute the six administrative units of Kyangwali, Kigorobya, Kabwoya Buseruka, Kigorobya town council and Kyangwali refugee settlement (Old and New) as shown in Figure 2.

Population and design for the Monitoring and Evaluation of the OCV Campaign. Two sub-studies were conducted to assess different aspects of the campaign. In sub-study one, a representative sample of the population that was targeted for vaccination was questioned to determine vaccine coverage rates, detect adverse events following immunization (AEFI) and collect additional information from the communities about the vaccine campaign. Sub-study two consisted of a survey among the campaign staff who participated in the OCV campaign after each round to assess their knowledge and practices.

For peer review only - http://bmjopen.bmj.com/site/about/guidelines.xhtml

Sub-study 1, community assessment. Sub-study one was a survey conducted in the vaccine target area, consisting of 31 clusters, each cluster consisting of 4 to 7 households per cluster. The study population included each person > one year of age who was living in the OCV targeted area at the time of the vaccination campaign. We assumed a household size of five persons based on estimates from a Demographic Health Survey conducted in 2016.[16] The sample was increased in order to raise the analytical power and precision of the surveys and to allow for separate analysis by gender. The formula used for determining sample size was, $n = (z^2pq)/d^2$,[17] where 'n' is the number of people desired for the survey, 'd' is the precision of the result, 'z' is the confidence limit, and 'p' and 'q' correspond to the proportion of persons in the population who are immunised and not immunised, respectively. We chose to use a low coverage of 50%. To identify the clusters, a list of villages was obtained from each of the four sub-counties targeted for vaccination. From these lists, the Excel random number generator (=RANDBETWEENBOTTOM, TOP) was used to select the 31 villages from which households were selected. From each selected village a list of households was obtained from village administrative leader (Local Council (LC) – 1: is the smallest recognised administrative unit in Uganda. It is headed an elected leader called LC-1) and used to identify the household by random selection similar to that used to select the villages or clusters. The sub-county population was obtained from the district planning unit and used to compute the number of households for each sub county as indicated in Table 1. The number of villages selected in each sub-county was in proportion to the population of the sub-county.

For the household interviews, data were collected through standardized questionnaires during face-to-face interviews conducted by trained research assistants using the local language. Within a selected household the questionnaires were administered to the key respondents (head of the HHs), who represented the entire household and provided information about each member of the household. If a suitable key respondent was absent, additional visits were scheduled. In two households, a person could not be located, and the household was dropped. For each vaccinated person, the research assistants assessed cholera immunization status. Vaccination status was ascertained in two ways: either the informant verbally indicated that the individual had received the dose of vaccine or the vaccination was recorded on the vaccine card. If the informant did not have a vaccine card, the reliability of the vaccination information was validated by asking about the details of the procedures of the vaccination (e.g. being given

For peer review only - http://bmjopen.bmj.com/site/about/guidelines.xhtml

recently by mouth to all persons > 1 year of age). None of the residents who were approached refused to answer the survey.

Adverse Events Following Immunization (AEFI). The occurrence of AEFIs were assessed by asking for symptoms among the vaccine recipients following vaccination. As part of the campaign itself, a routine system for AEFI detection was established in which the vaccine team members advised vaccinees to report to a health worker or to seek care at a health facility if they experienced symptoms following immunization. By contrast, the AEFI surveillance in this sub-study asked the participants who participated in the cluster survey about symptoms they may have experienced. This AEFI sub-study was thus, designed to enhance our understanding of potential AEFIs which may not have been reported through the routine AEFI surveillance.

AEFIs were categorised for each individual member of the household who received a dose of OCV as follows. They were considered mild if the symptoms did not interfere with normal activities; moderate if they interfered somewhat; and severe if the symptoms prevented the individual from continuing normal activities.[18] Persons who reported to be having ongoing symptoms > 72 hours were advised to visit the nearest health facility for more care. Among those reporting symptoms, information was recorded as to whether the person took any medicine or received any treatment to lessen the symptoms.

Data collection and analysis. Data from the community surveys were collected by tablet computers using *Kobo Collect* (https://www.kobotoolbox.org/) installed to record the responses in the field. Data were cleaned, coded, and stored in Stata Version 14. Data were analysed to generate frequencies, percentages or proportions and means. Comparisons between groups was done via logistic regression for the calculation of odds ratios and 95% confidence intervals. The results of analysis were presented in the form of graphs, tables, charts, and means and were included in interim and end of campaign reports.

Quality Assurance. Research assistants were trained on data collection methods and were able to consult field supervisors and the principal investigator on any issue that was not clear to them. For quality assurance, the survey supervisors revisited about 10% of the households, not to collect the data again, but to ensure that they were not skipped by the interviewers for eligible respondents. The surveys were conducted about two weeks after completion of the second round of the vaccination campaign to minimize recall bias.

For peer review only - http://bmjopen.bmj.com/site/about/guidelines.xhtml

Sub-study 2, staff assessment. Staff were assessed on their level of knowledge on the cause of cholera, the importance of safe water in cholera prevention, the target age group for cholera vaccination, and knowledge about AEFI and the procedures for care should subjects experience an AEFI. Staff were surveyed twice with each survey taking place within two weeks after administration of the first and second OCV rounds, respectively. All staff who participated directly by administering the vaccines or indirectly through supervisory roles and who were present at the workstation during the study period were enrolled in the survey. For the staff survey, structured questions were administered on paper questionnaires that allowed for adding text to explain the answers (open-ended questions). Most of the vaccine staff had taken part in other public health campaigns, but none had participated earlier with a campaign to distribute OCV.

Ethical Considerations. This study was conducted as part of the routine MoH operational research for improvement of health services; however, ethical issues were considered and addressed. The proposal was approved by the Makerere University School of Public Health Institutional Review Board (MaKSPH IRB) (no 610 in 2018) and Uganda National Council of Science and Technology. Written informed consent was obtained from all participants in both sub-studies. Participation in the study was voluntary and respondents were free to opt out at any stage of the interviews.

Confidentiality was observed at all stages of the study. No names or personal identifiers were included on the questionnaires. The research assistants underwent training on interview techniques, neutrality, and research ethics. The benefits of the study to the staff included the ability to express themselves, provide feedback and observations that in turn might lead to improvements in supportive services for their training and work.

Patient and Public Involvement. This research was done without research subject involvement. The time was inadequate to involve the subjects prior to the vaccine campaign. They were also not invited to contribute to the writing or editing of this document for readability or accuracy. However, the findings of the study were disseminated to the Hoima district administration, MoH and other policymakers to use them to strengthen health service interventions and future OCV campaigns.

RESULTS

For peer review only

For peer review only - http://bmjopen.bmj.com/site/about/guidelines.xhtml

Sub-study One, community survey results. The community surveys were carried out in the four sub-counties in Hoima districts of Buseruka, Kabwoya, Kyangwali and Kigorobya as shown in Figure 2 and Table 1. [Insert Figure 2 and Table 1] A total of 209 households, including 1,274 individuals, were surveyed. Most (96%) of the respondents were household heads or their spouses. All respondents confirmed that they were living in the targeted OCV area at the time of the campaign. Fifty-one (51%) of the respondents had primary education, 17% had secondary education, 1% had tertiary education, and the remaining 31% had no education. The respondents were aged 18 – 89 years with a mean age of 40 years. Both sexes were present, with no statistically significant difference.

By verbal reports, 94% of the residents received at least one dose and 78% received two doses of OCV. From verbal reporting, 91% of residents received vaccine during the first round and 81% received vaccine during the second round. For many of the households, a vaccine card was available, and the vaccination card was used to confirm vaccination status. Using information from the card only, coverage was 84% and 65% for round one and round two respectively and the two-dose coverage was 62% (95%CI: 59.4-64.9). Coverage rates are shown on Table 2. [Insert Table 2]

Among those who did not receive a dose of vaccine, over half of these missed doses (254 of the 357 missed doses during the two rounds) were because the person was not at home at the time of vaccination or was out of town. In a few cases, the vaccine team missed the household, accounting for 53 missed doses. Refusing to take vaccine was not reported.

Reported AEFIs. Overall, 71 individuals of 1,274 respondents (5.6%) reported an AEFI (Table 3). Determining a causal relation between the vaccination and the reported symptoms was not attempted. [Insert Table 3]

Most AEFIs were considered mild or moderate, but 8 (0.6%) persons reported an AEFI as severe. Most (60%) of the persons reporting an AEFI did not seek treatment including 60% of those reporting a severe AEFI. 29.6% of the reported adverse events occurred in the first round, 40.9% in the second round and 29.6% in both rounds. The most common symptoms were abdominal pain (15), diarrhoea, (9), fever, nausea, and headache (each 6) reports. Table 4 provides additional information on the AEFIs. The reported AEFIs were infrequent relative to the number of doses distributed and there were no serious adverse events reported. [Insert Table4]

For peer review only - http://bmjopen.bmj.com/site/about/guidelines.xhtml

Community knowledge of oral cholera vaccines.

A majority (77%) of the respondents understood that vaccine was one of the ways to prevent cholera. There was a statistically significant association between education level and knowledge about OCV with those having at least a primary school education being almost twice as likely to know the number of required doses as compared to those with no education (OR 1.90, 95% CI 1.06, 3.44 [P = 0.03].

Sub-study Two, staff survey. A total of 242 and 125 staff responded to the first and second knowledge and practice (KP) surveys (KP1 and KP2). Most respondents were vaccination team members (89% and 87% in vaccine rounds 1 and 2, respectively). Almost all the respondents were knowledgeable about the cause of cholera, the importance of safe water in cholera prevention and the vaccine target group, but were less knowledgeable regarding potential adverse events following administration (AEFI) or how to advise vaccinees, with 29% and 16% being less informed about AEFI during the first and second surveys.

When staff were asked to suggest areas that needed improvement in future OCV campaigns, more than 10% suggested more timely payment of allowances, more time to sensitize and inform the communities on the benefits of the vaccine, and better transportation and facilitation allowances (payments to health workers to cover the cost they incurred when administering the vaccines or conducting activities related to the OCV campaign). Other suggestions included use of both static and mobile vaccination points, provision of gumboots, umbrellas, and more areas for vaccine storage in sub-counties where vaccine would be more accessible, more workers for hard to reach areas, and an increase in the number of vaccine days to complete the vaccinations and increase coverage.

DISCUSSION

The results of this monitoring and evaluation exercise documented important findings on the OCV campaign, the knowledge and practices of both the community and the health staff involved in the campaign and implications for the conduct of future OCV campaigns as part of an integrated cholera control strategy. These findings suggest that the OCV campaign in Hoima successfully provided the vaccine to a very large proportion of the target population in Hoima district, western Uganda. Approximately 93.6% of respondents reported receiving at least one dose and 78.3% reported receiving two doses among residents. Given the mobile and transient nature of this population, this was noteworthy, and suggests that even better coverage may be possible for more settled populations in Uganda.

For peer review only - http://bmjopen.bmj.com/site/about/guidelines.xhtml

Since this was the first such campaign with OCV, there was concern that the population might be reluctant to accept it. This is a vaccine with which they were not familiar, it was given orally to all ages rather by injection to children, and two doses were required. Despite these potential constraints, we found that most people accepted taking the vaccine readily; however, some were not at home resulting in missed vaccinations.

High vaccination coverage is especially important when one is attempting to achieve herd protection. Since it is estimated that herd protection can be achieved with a coverage even lower than 90%;[19] the high coverage achieved in this campaign would be expected to induce significant indirect protection even among those who did not receive vaccine.[19 20]

It was noted in the administrative report from the MoH and during a stakeholder's meeting that one of the reasons for the reduction in the coverage during the second OCV dose was the unpredictable campaign dates for the second round. The vaccine for the second round had to be shipped and cleared through customs, and the timing for this was not certain. To avoid this problem in the future, a mechanism needs to be established to provide a better timeline for receipt of the vaccine shipments.

Community Reception to OCV. As with previous OCV campaigns outside Uganda, very few AEFI were reported.[21 22] Most of adverse events were considered mild or moderate and were self-limited. Despite the low prevalence of AEFI, the survey exposed the need to better inform the community about seeking treatment for more severe adverse events or for those that do not quickly resolve. This was especially true for families with little education who were less likely to seek medical attention for severe AEFI (data not shown). Notably, members in the community demonstrated good understanding of the rationale for the vaccine; however, a key takeaway from the survey was a need to better communicate the number of required doses, given that those with more education were twice as likely than those with no education to know the number of doses needed.

Staff Reception to OCV. Inclusion of staff KP survey contributed to the success of the project by identifying gaps among the staff knowledge and performance. Questioning the vaccine staff about their training and their experience in the field is not a common activity when conducting monitoring and evaluation activities during OCV campaigns. Many people had to be mobilized quickly and these were the key people who interacted with the communities. It was important

For peer review only - http://bmjopen.bmj.com/site/about/guidelines.xhtml

that the staff accurately represent the campaign as an integrated cholera prevention program, but this was the first time these people carried out this role. The MoH felt it important to monitor their knowledge and behaviors as well as any constraints they felt in carrying out their functions. While they were generally knowledgeable about the disease and about the vaccine, these staff needed additional training regarding recognizing and managing AEFIs. They also faced challenges regarding logistical support. After the first staff KP survey, these gaps were communicated to the MoH so that appropriate actions could be taken to ensure that these gaps were addressed prior to the second round.

Most other OCV campaigns have also reported high coverage rates. These have included reports from Bangladesh[23 24], Malawi[25 26], Mozambique[21],Democratic Republic of Congo [27], Zambia [28], South Sudan [29 30], Iraq[31], Haiti[32], and Guinea [33]. Clearly, OCV is well accepted among these very diverse population groups where the vaccine campaigns have been carried out.

Important limitations of this study need to be mentioned. Ideally, one would prefer to conduct community studies prior to a campaign to understand knowledge and attitudes about cholera to improve communications regarding the upcoming campaign as part of an integrated strategy to control cholera. However, since the campaign was carried out on an emergency basis during an outbreak, a study prior to the campaign was not possible. Secondly, a community survey immediately after the first round might have provided feedback to the teams that would have improved the coverage for the second round. It should also be noted that a single informant provided information about receipt of the vaccine for all members of the household, so this informant might have incorrect information concerning one or more members of the household; however, since the vaccine was directly given to the household members together, it seems that inaccuracies would be minimal. The community KP survey did not include questions on attitudes regarding cholera. Since the survey had to be carried out very quickly following the campaign, and since the survey was targeted to identify issues that would be immediately relevant to campaign performance, it was felt that understanding attitudes regarding cholera, even though important, would have required other qualitative methods requiring more time than was available. Similarly, direct observation of the training and coordination meetings would have been useful to independently assess the efficiency and effectiveness of these training and coordination meetings. Furthermore, there was no list of all workers in the campaign and many of the workers who participated in the second round had left prior to administering the

For peer review only - http://bmjopen.bmj.com/site/about/guidelines.xhtml
For peer review only

questionnaire; thus, there were fewer respondents in the second round and the proportion of all workers who participated could not be determined precisely. Finally, it was not possible, given the time constraints, to fully integrate WASH interventions together with the OCV campaign, or to monitor and evaluate community and staff responsiveness to such integration.

In this outbreak 2,122 cholera cases and 44 deaths were reported, nearly all before the OCV campaign and over half occurred in the first two weeks of the outbreak. Of note, the outbreak started in February 2018 at about the same time the application for preventive use of OCV was being was submitted. The original application proposed a series of preventive campaigns over the next year, and Hoima, as well as neighbouring districts in western Uganda, were targeted for vaccination in the first round of these preventive campaigns. However, when the outbreak was identified, plans were quickly shifted so that an emergency campaign could be implemented to control the outbreak. Even though this emergency response was planned as quickly as possible, in fact, the outbreak was essentially over before the vaccine campaign could start, so it had no impact on the outbreak itself, but likely reduced the risk for future outbreaks.

Though the outbreak started with the influx of the refugees from DRC into Uganda, it quickly spread to the refugee host communities in Hoima. Therefore, to prevent rapid spread, improvement of cholera prevention measures for both the refugees and the host communities is paramount during resettlement.

CONCLUSION

This study suggests that the OCV campaign in Hoima district to prevent cholera was successful and achieved a high level of coverage in this population that was targeted to be at risk. However, there was need to devote more effort on community sensitisation on the benefits of vaccination, as well as improving some logistic support during the campaign.

While a rapid response to this outbreak was appropriate, in fact, even with a rapidly organized campaign, the outbreak was over before the vaccine could be given; thus, the vaccine had no impact on this outbreak. Nevertheless, this area had already been identified as a hotspot, and it would have been targeted if the planned preventive campaign had proceeded as originally planned. Planners must realize that an area identified as a hotspot might experience an

For peer review only - http://bmjopen.bmj.com/site/about/guidelines.xhtml

outbreak while preparations are underway for a preventive campaign and take this into account when allocating vaccine for preventive vs emergency campaigns. Since these are areas where cholera risk is high, outbreaks are likely to occur in these areas if there are delays in implementing preventive campaigns.

For peer review only

For peer review only - http://bmjopen.bmj.com/site/about/guidelines.xhtml

447 **FIGURE LEGENDS**

449 **Figure 1. Epicurve of the Hoima Outbreak, 2018 with events identified in response to the outbreak**

451 **Figure 2: Map of Hoima District, showing sub-counties that received OCV and households where interviews were conducted (red dots)**

For peer review only

For peer review only - http://bmjopen.bmj.com/site/about/guidelines.xhtml

Table 1. Age and Sex Breakdown of the Participants in the Cluster Survey

Age Group (years)	Male	Female	Total
**1 to 4**	98	93	191
**5 to 14**	236	207	443
**15 to 44**	210	285	495
**45+**	80	65	145
**Total**	625	650	1274

Table 2: Vaccination coverage post OCV campaign, Hoima District, Uganda, 2018.

Total surveyed = 1274	Round 1	Round 2	Received only one dose	Received two doses	Received at least one dose
**Reported number (%) (95% CI)**	1,164 (91.4) (89.7-92.8)	1,027 (80.6) (78.3-82.7)	195 (15.3) (13.4-17.4)	998 (78.3) (76.0-80.5)	1193 (93.6) (92.2-94.9)
**Confirmed by availability of the vaccination card (%) (95% CI)**	1,065 (83.6) (81.5-85.5)	823 (64.6) (61.9-67.2)	142 (11.1) (9.5-13.0)	792 (62.2) (59.5-64.8)	934 (73.3) (70.8-75.7)

Table 3: Treatment and Resolution of Adverse Events Following Immunization in Hoima District, Uganda, 2018.

Symptoms	Treatment		Status		
	**No treatment (%)**	**Treated (%)**	**Recovered (%)**	**Ongoing (%)**	**Improved, not to baseline (%)**
**Mild**	24(80.0)	6(20.0)	29(96.7)	1(3.3)	0(0.0)
**Moderate**	11(39.3)	17(60.7)	23(82.1)	3(10.7)	2(7.1)
**Severe**	8(61.5)	5(38.5)	10(76.9)	0(0.0)	3(23.1)

For peer review only

For peer review only - http://bmjopen.bmj.com/site/about/guidelines.xhtml

Table 4. Onset and frequency of symptoms reported as adverse events

	<6 hours	6-12 hours	12-24 hours	1-7 days	8-14 days	Total
**Diarrhoea**	3	2	2	2	0	9
**Vomiting**	3	0	0	2	0	5
**Nausea**	6	0	0	0	0	6
**Abdominal pain**	15*	0	0	0	0	15
**Stomach gurgling**	0	3	0	1	0	4
**Mouth ulcers**	0	0	0	0	1	1
**Cough**	1	1	0	3	1	6
**Felt feverish**	1	1	0	6	1	9
**Poor appetite**	1	0	0	0	0	1
**Dizziness**	0	3	0	0	0	3
**Fainted**	0	1	0	0	0	1
**Itching**	0	0	0	0	1	1
**Weakness**	0	0	0	1	0	1
**Headache**	3	0	2	1	0	6
**Other**	1	0	0	2	0	3
**Total**	34	11	4	18	4	71

- **Three persons reported abdominal pain in one household**

For peer review only

Acknowledgements

The authors thank the persons and institutions who supported Hoima district OCV monitoring and evaluation study. Special thanks are extended to the research assistants who dedicated their time to meticulously collect the data and the persons who collaborated in the preparation of the different key documents used in this study. We thank the staff, the communities in the study area and the local leadership of Hoima districts for the cooperation during this study. We are grateful to Ambrose Buyinza Wabwire for technical support with drawing of the maps.

We thank the Ministry of Health leadership for guidance; the Office of the Prime Minister (OPM) for the permission to access data on the refugee population. We are grateful to the following institutions and bodies; Ministry of Health, Makerere University School of Public Health, World Health Organisation, UNICEF and UNHCR for technical guidance.

Footnotes

Contributors: Conceived and designed the study: GB, MR, WAB, ABS, CGO, DAS. Discussed, critically revised and approved the study protocol: GB, MR, WAB, CGO, DAS. Performed the research: GB, MR, WAB, AO, FR, IA. Analysed the data: GB, MR, WAB, AO, DAS. Wrote the first draft: GB, MR. Wrote the final DAS. Elaborated, discussed and approved the final version: GB, MR, AB, ABS, AO, FR, IA, CGO, and DAS.

Funding: Funding for the study was provided by the Bill and Melinda Gates Foundation (OPP1148763) which provided financial support through the John Hopkins University, Delivery of Oral Cholera Vaccine Effectively (DOVE) project. The funding agency had no role in collecting, analysing or interpreting the results.

Declaration of the conflict of interest

The authors have no conflict of interest to declare.

Additional data sharing: No additional data available.

For peer review only - http://bmjopen.bmj.com/site/about/guidelines.xhtml

REFERENCES

1. Clemens JD, Nair GB, Ahmed T, et al. Cholera. *Lancet* 2017 doi: 10.1016/S0140-6736(17)30559-7
2. Cholera vaccines: WHO position paper - August 2017. *Wkly Epidemiol Rec* 2017;92(34):477-98.
3. Kirigia JM, Sambo LG, Yokouide A, et al. Economic burden of cholera in the WHO African region. *BMC Int Health Hum Rights* 2009;9:8. doi: 10.1186/1472-698X-9-8
4. Lessler J, Moore SM, Luquero FJ, et al. Mapping the burden of cholera in sub-Saharan Africa and implications for control: an analysis of data across geographical scales. *Lancet* 2018;391(10133):1908-15. doi: 10.1016/S0140-6736(17)33050-7
5. Bwire G, Ali M, Sack DA, et al. Identifying cholera "hotspots" in Uganda: An analysis of cholera surveillance data from 2011 to 2016. *PLoS Negl Trop Dis* 2017;11(12):e0006118. doi: 10.1371/journal.pntd.0006118
6. Cummings MJ, Wamala JF, Eyura M, et al. A cholera outbreak among semi-nomadic pastoralists in northeastern Uganda: epidemiology and interventions. *Epidemiol Infect* 2012;140(8):1376-85. doi: 10.1017/S0950268811001956 [published Online First: 2011/09/29]
7. Bwire G, Mwesawina M, Baluku Y, et al. Cross-Border Cholera Outbreaks in Sub-Saharan Africa, the Mystery behind the Silent Illness: What Needs to Be Done? *PLoS One* 2016;11(6):e0156674. doi: 10.1371/journal.pone.0156674
8. Bwire G, Malimbo M, Maskery B, et al. The burden of cholera in Uganda. *PLoS Negl Trop Dis* 2013;7(12):e2545. doi: 10.1371/journal.pntd.0002545
9. Oguttu DW, Okullo A, Bwire G, et al. Cholera outbreak caused by drinking lake water contaminated with human faeces in Kaiso Village, Hoima District, Western Uganda, October 2015. *Infect Dis Poverty* 2017;6(1):146. doi: 10.1186/s40249-017-0359-2
10. Alajo SO, Nakavuma J, Erume J. Cholera in endemic districts in Uganda during El Nino rains: 2002-2003. *Afr Health Sci* 2006;6(2):93-7. doi: 10.5555/afhs.2006.6.2.93 [published Online First: 2006/08/19]
11. Montgomery M, Jones MW, Kabole I, et al. No end to cholera without basic water, sanitation and hygiene. *Bull World Health Organ* 2018;96(6):371-71A. doi: 10.2471/BLT.18.213678
12. Legros D, Partners of the Global Task Force on Cholera C. Global Cholera Epidemiology: Opportunities to Reduce the Burden of Cholera by 2030. *J Infect Dis* 2018 doi: 10.1093/infdis/jiy486
13. Deployments from the oral cholera vaccine stockpile, 2013-2017. *Wkly Epidemiol Rec* 2017;92(32):437-42.
14. Odevall L, Hong D, Digilio L, et al. The Euvichol story - Development and licensure of a safe, effective and affordable oral cholera vaccine through global public private partnerships. *Vaccine* 2018;36(45):6606-14. doi: 10.1016/j.vaccine.2018.09.026
15. Bwire G, Munier A, Ouedraogo I, et al. Epidemiology of cholera outbreaks and socio-economic characteristics of the communities in the fishing villages of Uganda: 2011-2015. *PLoS Negl Trop Dis* 2017;11(3):e0005407. doi: 10.1371/journal.pntd.0005407

For peer review only

For peer review only - http://bmjopen.bmj.com/site/about/guidelines.xhtml

16. Uganda_Bureau_of_Statistics_(UBOS). Demographic and Health Survey. 2016 Kampala and Rockville Maryland2016 [Available from: https://www.dhsprogram.com/pubs/pdf/FR333/FR333.pdf accessed Sept 18. 2020 2020.
17. Henderson RH, Sundaresan T. Cluster sampling to assess immunization coverage: a review of experience with a simplified sampling method. *Bull World Health Organ* 1982;60(2):253-60.
18. Food_and_Drug_Administration. Guidance for Industry. Toxicity Grading Scale for Healthy Adult and Adolescent Volunteers Enrolled in Preventive Vaccine Clinical Trials Rockville, Maryland USA: Food and Drug Administration, Center for Biologics Evaluation and Research 2007 [cited 2020. Available from: https://www.fda.gov/media/73679/download accessed September 22 2020.
19. Longini IM, Jr., Nizam A, Ali M, et al. Controlling endemic cholera with oral vaccines. *PLoS Med* 2007;4(11):e336. doi: 10.1371/journal.pmed.0040336 [published Online First: 2007/11/30]
20. Ali M, Emch M, von Seidlein L, et al. Herd immunity conferred by killed oral cholera vaccines in Bangladesh: a reanalysis. *Lancet* 2005;366(9479):44-9.
21. Sema Baltazar C, Rafael F, Langa JPM, et al. Oral cholera vaccine coverage during a preventive door-to-door mass vaccination campaign in Nampula, Mozambique. *PLoS One* 2018;13(10):e0198592. doi: 10.1371/journal.pone.0198592
22. Phares CR, Date K, Travers P, et al. Mass vaccination with a two-dose oral cholera vaccine in a long-standing refugee camp, Thailand. *Vaccine* 2016;34(1):128-33. doi: 10.1016/j.vaccine.2015.10.112
23. Khan AI, Khan IA, Siddique SA, et al. Feasibility, coverage and cost of oral cholera vaccination conducted by icddr,b using the existing national immunization service delivery mechanism in rural setting Keraniganj, Bangladesh. *Hum Vaccin Immunother* 2019;15(6):1302-09. doi: 10.1080/21645515.2018.1528833
24. Uddin MJ, Wahed T, Saha NC, et al. Coverage and acceptability of cholera vaccine among high-risk population of urban Dhaka, Bangladesh. *Vaccine* 2014;32(43):5690-5. doi: 10.1016/j.vaccine.2014.08.021
25. Grandesso F, Rafael F, Chipeta S, et al. Oral cholera vaccination in hard-to-reach communities, Lake Chilwa, Malawi. *Bull World Health Organ* 2018;96(12):817-25. doi: 10.2471/BLT.17.206417
26. Sauvageot D, Saussier C, Gobeze A, et al. Oral cholera vaccine coverage in hard-to-reach fishermen communities after two mass Campaigns, Malawi, 2016. *Vaccine* 2017;35(38):5194-200. doi: 10.1016/j.vaccine.2017.07.104
27. Massing LA, Aboubakar S, Blake A, et al. Highly targeted cholera vaccination campaigns in urban setting are feasible: The experience in Kalemie, Democratic Republic of Congo. *PLoS Negl Trop Dis* 2018;12(5):e0006369. doi: 10.1371/journal.pntd.0006369
28. Poncin M, Zulu G, Voute C, et al. Implementation research: reactive mass vaccination with single-dose oral cholera vaccine, Zambia. *Bull World Health Organ* 2018;96(2):86-93. doi: 10.2471/BLT.16.189241

For peer review only - http://bmjopen.bmj.com/site/about/guidelines.xhtml

29. Parker LA, Rumunu J, Jamet C, et al. Neighborhood-targeted and case-triggered use of a single dose of oral cholera vaccine in an urban setting: Feasibility and vaccine coverage. *PLoS Negl Trop Dis* 2017;11(6):e0005652. doi: 10.1371/journal.pntd.0005652
30. Porta MI, Lenglet A, de Weerdt S, et al. Feasibility of a preventive mass vaccination campaign with two doses of oral cholera vaccine during a humanitarian emergency in South Sudan. *Trans R Soc Trop Med Hyg* 2014;108(12):810-5. doi: 10.1093/trstmh/tru153
31. Lam E, Al-Tamimi W, Russell SP, et al. Oral Cholera Vaccine Coverage during an Outbreak and Humanitarian Crisis, Iraq, 2015. *Emerg Infect Dis* 2017;23(1):38-45. doi: 10.3201/eid2301.160881
32. Tohme RA, Francois J, Wannemuehler K, et al. Oral Cholera Vaccine Coverage, Barriers to Vaccination, and Adverse Events following Vaccination, Haiti, 2013. *Emerg Infect Dis* 2015;21(6):984-91. doi: 10.3201/eid2106.141797
33. Luquero FJ, Grout L, Ciglenecki I, et al. First outbreak response using an oral cholera vaccine in Africa: vaccine coverage, acceptability and surveillance of adverse events, Guinea, 2012. *PLoS Negl Trop Dis* 2013;7(10):e2465. doi: 10.1371/journal.pntd.0002465

For peer review only

For peer review only - http://bmjopen.bmj.com/site/about/guidelines.xhtml

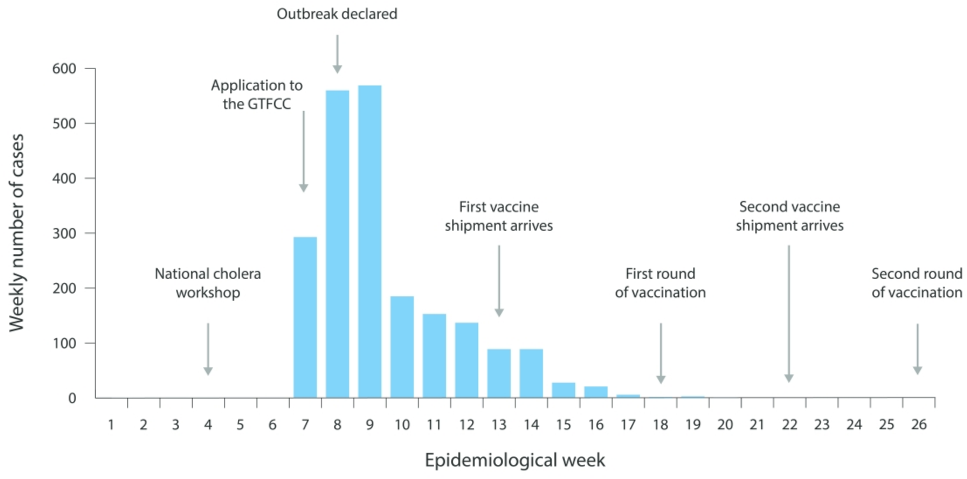

Figure 1. Epicurve of the Hoima Outbreak, 2018 with events identified in response to the outbreak

542x305mm (300 x 300 DPI)

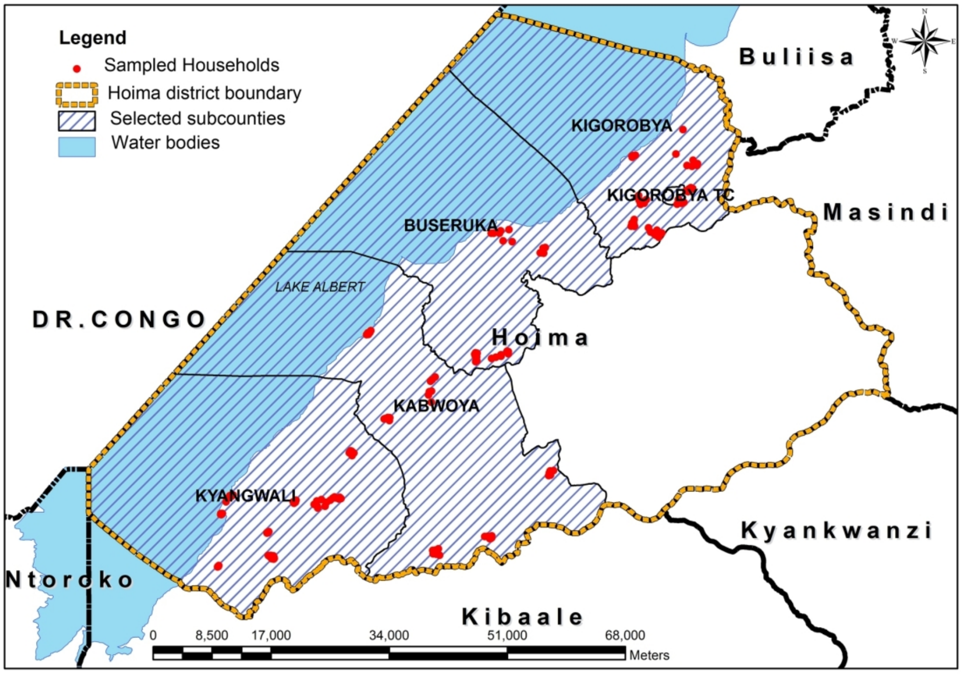

Figure 2: Map of Hoima District, showing sub-counties that received OCV and households where interviews were conducted (red dots)

170x119mm (300 x 300 DPI)

BMJ Open

Use of surveys to evaluate an integrated oral cholera vaccine campaign in response to a cholera outbreak in Hoima District, Uganda

Journal:	*BMJ Open*
Manuscript ID	bmjopen-2020-038464.R2
Article Type:	Original research
Date Submitted by the Author:	28-Oct-2020
Complete List of Authors:	Bwire, Godfrey; Republic of Uganda Ministry of Health, Community Health<br>Roskosky, Mellisa; Johns Hopkins University Bloomberg School of Public Health, International Health<br>Ballard, Anne; Johns Hopkins University Bloomberg School of Public Health, Center for Communications Programs<br>Brooks, W; Johns Hopkins University Bloomberg School of Public Health, International Health<br>Alfred, Okello; Makerere University College of Health Sciences<br>Rafael, Florentina; World Health Organization, Infectious Hazard Management<br>Ampeire, Immaculate; Republic of Uganda Ministry of Health, Expanded Programme on Immunization<br>Orach, Christopher; Makerere University College of Health Sciences<br>Sack, David; Johns Hopkins University Bloomberg School of Public Health, International Health
**Primary Subject Heading**:	Global health
Secondary Subject Heading:	Epidemiology, Health services research, Infectious diseases, Public health
Keywords:	Gastrointestinal infections < GASTROENTEROLOGY, International health services < HEALTH SERVICES ADMINISTRATION & MANAGEMENT, Epidemiology < INFECTIOUS DISEASES, Infection control < INFECTIOUS DISEASES, Public health < INFECTIOUS DISEASES, Tropical medicine < INFECTIOUS DISEASES

SCHOLARONE™
Manuscripts

I, the Submitting Author has the right to grant and does grant on behalf of all authors of the Work (as defined in the below author licence), an exclusive licence and/or a non-exclusive licence for contributions from authors who are: i) UK Crown employees; ii) where BMJ has agreed a CC-BY licence shall apply, and/or iii) in accordance with the terms applicable for US Federal Government officers or employees acting as part of their official duties; on a worldwide, perpetual, irrevocable, royalty-free basis to BMJ Publishing Group Ltd ("BMJ") its licensees and where the relevant Journal is co-owned by BMJ to the co-owners of the Journal, to publish the Work in this journal and any other BMJ products and to exploit all rights, as set out in our licence.

The Submitting Author accepts and understands that any supply made under these terms is made by BMJ to the Submitting Author unless you are acting as an employee on behalf of your employer or a postgraduate student of an affiliated institution which is paying any applicable article publishing charge ("APC") for Open Access articles. Where the Submitting Author wishes to make the Work available on an Open Access basis (and intends to pay the relevant APC), the terms of reuse of such Open Access shall be governed by a Creative Commons licence – details of these licences and which Creative Commons licence will apply to this Work are set out in our licence referred to above.

Other than as permitted in any relevant BMJ Author's Self Archiving Policies, I confirm this Work has not been accepted for publication elsewhere, is not being considered for publication elsewhere and does not duplicate material already published. I confirm all authors consent to publication of this Work and authorise the granting of this licence.

For peer review only

Use of surveys to evaluate an integrated oral cholera vaccine campaign in response to a cholera outbreak in Hoima District, Uganda

Godfrey Bwire[1,] Mellisa Roskosky[2], Anne Ballard Sara[2], W. Abdullah Brooks[2], Alfred Okello[3], Florentina Rafael [4], Immaculate Ampeire[5], Christopher Garimoi Orach[3], David A. Sack[2].

Authors' affiliations:

1. Ministry of Health, Department of Community Health, Kampala Uganda.
2. Johns Hopkins Bloomberg School of Public Health, Department of International Health, Baltimore, Maryland USA.
3. Makerere University, College of Health Sciences, Kampala, Uganda.
4. World Health Organization, Department of Infectious Hazard Management, Geneva, Switzerland.
5. Ministry of Health, Uganda National Expanded Program on Immunization

*Corresponding author.

Professor David A. Sack, M, D.

Johns Hopkins Bloomberg School of Public Health

615 North Wolfe Street

Baltimore, Maryland 21205 USA

Phone +1 443 287 8795

Email: dsack1@jhu.edu

Number of words (Excluding abstract): 4,735
Number of words in abstract: 264
Number of tables: 4
Number of figures: 2
Number of references: 33
Number of supplemental materials: 0

Key words: cholera, vaccination, Uganda, monitoring, coverage, outbreak, sub-Saharan Africa, oral cholera vaccine, Vaccine campaign

For peer review only - http://bmjopen.bmj.com/site/about/guidelines.xhtml

ABSTRACT

Objectives To evaluate the quality and coverage of the campaign to distribute oral cholera vaccine during a cholera outbreak in Hoima, Uganda to guide future campaigns of cholera vaccine.

Design Survey of communities targeted for vaccination to determine vaccine coverage rates and perceptions of the vaccination campaign, and a separate survey of vaccine staff who carried out the campaign.

Setting Hoima District, Uganda.

Participants Representative clusters of households residing in the communities targeted for vaccination and staff members who conducted the vaccine campaign.

Results Among 209 households (1,274 individuals) included in the coverage survey, 1193 (94%; 95% CI: 92-95%) reported receiving at least one OCV dose and 998 (78%; 95% CI: 76-81%) reported receiving two doses. Among vaccinated individuals, minor complaints were reported by 71 persons (5.6%). Individuals with 'some' education (primary school or above) were more knowledgeable regarding the required OCV doses compared to non-educated (p value = 0.03). Factors negatively associated with campaign implementation included community sensitisation time, staff payment, and problems with field transport. Although the campaign was carried out quickly, the outbreak was over before the campaign started. Most staff involved in the campaign (93%) were knowledgeable about cholera control; however, 29% did not clearly understand how to detect and manage adverse events following immunization.

Conclusion The campaign achieved high OCV coverage, but the surveys provided insights for improvement. To achieve high vaccine coverage, more effort is needed for community sensitisation, and additional resources for staff transportation and timely payment for campaign staff is required. Pre and post-test assessment of staff training can identify and address knowledge and skill gaps.

For peer review only

Strengths and limitations of this study

- The cluster survey of households in communities targeted for vaccination efficiently documented actual vaccine coverage in the target population.
- The cluster surveys of households identified mild adverse events not identified during the campaign and identified the emphasize the second dose, especially among less educated groups.
- Surveys of the vaccination staff immediately following each round identified certain weaknesses in staff orientation as well as constraints to their job performance in the field.
- The household surveys obtained data from a single spokesperson for the household rather than from each individual which might have introduced some uncertainty in the household data.
- Evaluation of the vaccination staff was carried through surveys and would have benefited by direct observation of the training and the field performance.

For peer review only

INTRODUCTION

Cholera, a preventable and treatable disease is characterized by profuse watery diarrhoea caused by infection of the intestine with the bacterium *Vibrio cholerae.*[1] Cholera is a major cause of morbidity and mortality in several countries in sub-Saharan Africa where cholera outbreaks also negatively affect development due to associated high economic burden. [2 3] Between 2010 and 2016 an average 141,918 incident cases annually were reported from sub-Saharan African countries, including Uganda.[4] In Uganda, cholera outbreaks occurred as both endemic and epidemic disease. Epidemic disease occurred in northern and eastern Uganda districts [5] and are thought to be worsened by contamination of water due to poor sanitation.[6] Cholera outbreaks especially occur in districts along the international borders with the Democratic Republic of the Congo (DR Congo), South Sudan and Kenya and along the Great Lakes.[5 7] These districts include Hoima, where cholera is endemic. [5 8-10]

There has been debate in the public health community on best practices for endemic and epidemic cholera disease control, with some preferring to focus on WASH interventions, and others advocating for OCV for both endemic and epidemic disease control.[11]. In part, this has been facilitated by a relative lack of experience with OCV and concern that excess reliance on vaccine might negatively affect essential infrastructural development and hygienic practices. The World Health Organization (WHO) recommends an integrated approach to cholera prevention where water, sanitation and hygiene (WaSH) interventions are complemented by vaccine campaigns which provide oral cholera vaccine (OCV) to persons living in areas considered high risk.[2 12] These vaccine campaigns may be either preventive, in which the vaccine is targeted to cholera hotspots, or reactive in which the campaign is implemented in response to an outbreak or a humanitarian emergency.[13]

Two WHO-prequalified currently OCVs are available from the global stockpile: Shanchol (Shantha Biotechnics Limited, India) and Euvichol (Eubiologics Co., Ltd., Korea).[2] The standard immunization schedule consists of two doses given at an interval of at least two weeks to all persons in the target area above one year of age. While there is increasing use of OCV to control outbreaks, preventive use is constrained due to inadequate vaccine supply.[14] Since creation of a global OCV stockpile in July, 2013, several OCV campaigns had been successfully implemented[13 14] but it is still important to document national campaign experiences as well as monitoring and evaluation activities, to continually improve the effectiveness and efficiency of vaccine campaigns.

For peer review only - http://bmjopen.bmj.com/site/about/guidelines.xhtml

The Ugandan Ministry of Health (MoH) had prepared plans for OCV campaigns in the areas identified as cholera hotspots starting in the western districts of Uganda (including Hoima), near the border with DR Congo and close to, or adjoining Lake Albert. These hotspot districts and their specified sub-counties were confirmed during a national cholera workshop in Kampala on 29-31 January 2018. This workshop led to the development of an application for OCV to the Global Taskforce for Cholera Control (GTFCC) which was submitted on 14 February 2018. The application proposed providing OCV in these identified hotspots as a preventive strategy. However, while preparations for these campaigns were underway, an outbreak was declared in Hoima district on 23 February 2018. The earliest cases were identified among DR Congo refugees, but then other cases were seen among the non-refugee Ugandan population. The MoH responded to the outbreak with multisectoral interventions, including proper case management, promotion of access to safe water and improved sanitation (WaSH), enhanced cholera surveillance, as well as infection control and health education. These measures were then supplemented with plans for an emergency OCV campaign. Thus, the original plans for a preventive OCV campaign were shifted to an emergency response to control the outbreak. The first doses of vaccine arrived on 28 March and the first round of vaccinations started on 2 May. The doses for the second round arrived on 29 May and the second round started on 26 June. A door-to-door strategy was used to deliver two doses of vaccine to an estimated 360,000 people, including pregnant women, over the age of one year residing in the four targeted sub-counties. To carry out the campaign, the MoH organized all activities including logistics, community mobilisation and implementation, coordinating ground activities through an assigned point person. Many stakeholders contributed to the campaign including the Hoima district local government, WHO, UNICEF, UNHCR and Médecins sans Frontiers (MSF). Prior to the campaign, the stakeholders met to define and coordinate their complementary tasks.

The epidemic curve based on a line list of cases and deaths by date and stated nationality is shown on Figure 1. Over the course of the outbreak, 2,122 cases with 44 deaths (case fatality rate, 2.1%) were reported. Sixty six percent (1,410) of the cases and 64% (28) of the deaths occurred during the first two weeks of the outbreak. Many of the cases and deaths (1276 and 32, or 60% and 73%, respectively) occurred among persons who were from DR Congo, and the refugees developed cholera symptoms soon after arrival in Uganda. Among the 44 deaths reported, 25 (57%) occurred in the community, not in the health facility. Nineteen of the fatal cases were treated at the health facility; the case fatality ratio (CFR) for facility-treated patients was 0.9%. Although the emergency vaccination campaign intended to control the outbreak,

For peer review only - http://bmjopen.bmj.com/site/about/guidelines.xhtml

because the outbreak was so sudden and so short-lived, the campaign could only be initiated after the outbreak had already declined.

Rationale. While vaccines are commonly used in Uganda, especially through the longstanding EPI program (Expanded Programme on Immunization), this was the first OCV use in Uganda, and there was no prior experience to guide responders and implementers. Thus, this study was carried out with the aim to document campaign activities and to monitor and evaluate its procedures and outcomes that could guide future OCV campaigns. The issues addressed during this study included the knowledge and practices of the campaign staff, vaccine coverage in the targeted areas, and the knowledge and practices of the community. After this initial campaign, the MoH continued its plans for preventive campaigns in the remaining cholera hotspot districts, informed by the lessons learned from this initial emergency use campaign. In an effort to document the impact of OCV on an outbreak in a setting with endemic disease, we undertook this monitoring and evaluation exercise, as described below.

MATERIALS AND METHODS

Study setting. Hoima district is located in western Uganda, across Lake Albert from DR Congo. It has a total area of 5735.5 square kilometres and a projected population of 630,000 persons (2018). The district consists of 13 administrative units as follows: 10 sub-counties (Kyabigambire, Buhimba, Kyangwali, Kabwoya, Bugambe, Kiziranfumbi, Kitoba, Kigorobya, Buseruka and Buhanika), a municipality (Hoima municipality) and two town councils (Kigorobya and Buhima town councils). The major economic activities of the population in Hoima are subsistence agriculture and fishing. Cholera is endemic in the district but the endemicity is localised in some specific sub-counties particularly those with fishing communities.[15] The sub-counties targeted for OCV included Buseruka, Kabwoya, Kangwali, and Kigorobya which together constitute the six administrative units of Kyangwali, Kigorobya, Kabwoya Buseruka, Kigorobya town council and Kyangwali refugee settlement (Old and New) as shown in Figure 2.

Population and design for the Monitoring and Evaluation of the OCV Campaign. Two sub-studies were conducted to assess different aspects of the campaign. In sub-study one, a representative sample of the population that was targeted for vaccination was questioned to determine vaccine coverage rates, detect adverse events following immunization (AEFI) and collect additional information from the communities about the vaccine campaign. Sub-study two consisted of a survey among the campaign staff who participated in the OCV campaign after each round to assess their knowledge and practices.

For peer review only - http://bmjopen.bmj.com/site/about/guidelines.xhtml

Sub-study 1, community assessment. Sub-study one was a two-stage, cluster survey conducted in the vaccine target area, consisting of 31 clusters, each cluster consisting of 4 to 7 households per cluster. The study population included each person > one year of age who was living in the OCV targeted area at the time of the vaccination campaign. We assumed a household size of five persons based on estimates from a Demographic Health Survey conducted in 2016.[16] The sample was increased in order to raise the analytical power and precision of the surveys and to allow for separate analysis by gender. The formula used for determining sample size was, $n = (z^2pq)/d^2$,[17] where 'n' is the number of people desired for the survey, 'd' is the precision of the result, 'z' is the confidence limit, and 'p' and 'q' correspond to the proportion of persons in the population who are immunised and not immunised, respectively. We chose to use a low coverage of 50%.

To identify the clusters, a list of villages was obtained from each of the four sub-counties targeted for vaccination. From these lists, the Excel random number generator (=RANDBETWEENBOTTOM, TOP) was used to select the 31 villages from which households were selected. The number of villages per sub-county was proportionate to the population of the sub-county. The sub-county populations were obtained from the district planning unit. From each selected village a list of households was obtained from the village administrative leader (Local Council (LC) – 1. This is the smallest recognised administrative unit in Uganda. It is headed an elected leader called LC-1) who provided a list of households from which we randomly selected households to interview.

For the household interviews, data were collected through standardized questionnaires during face-to-face interviews conducted by trained research assistants using the local language. Within a selected household the questionnaires were administered to the key respondents (head of the HHs), who represented the entire household and provided information about each member of the household. If a suitable key respondent was absent, additional visits were scheduled. In two households, a person could not be located, and the household was dropped. For each vaccinated person, the research assistants assessed cholera immunization status. Vaccination status was ascertained in two ways: either the informant verbally indicated that the individual had received the dose of vaccine or the vaccination was recorded on the vaccine card. If the informant did not have a vaccine card, the reliability of the vaccination information was validated by asking about the details of the procedures of

For peer review only - http://bmjopen.bmj.com/site/about/guidelines.xhtml

the vaccination (e.g. being given recently by mouth to all persons > 1 year of age). None of the residents who were approached refused to answer the survey. The age and sex of the participants in the survey is shown in Table 1.

Adverse Events Following Immunization (AEFI). The occurrence of AEFIs were assessed by asking for symptoms among the vaccine recipients following vaccination. As part of the campaign itself, a routine system for AEFI detection was established in which the vaccine team members advised vaccinees to report to a health worker or to seek care at a health facility if they experienced symptoms following immunization. By contrast, the AEFI surveillance in this sub-study asked the participants who participated in the cluster survey about symptoms they may have experienced. This AEFI sub-study was thus, designed to enhance our understanding of potential AEFIs which may not have been reported through the routine AEFI surveillance.

AEFIs were categorised for each individual member of the household who received a dose of OCV as follows. They were considered mild if the symptoms did not interfere with normal activities; moderate if they interfered somewhat; and severe if the symptoms prevented the individual from continuing normal activities.[18] Persons who reported to be having ongoing symptoms > 72 hours were advised to visit the nearest health facility for more care. Among those reporting symptoms, information was recorded as to whether the person took any medicine or received any treatment to lessen the symptoms.

Data collection and analysis. Data from the community surveys were collected by tablet computers using *Kobo Collect* (https://www.kobotoolbox.org/) installed to record the responses in the field. Data were cleaned, coded, and stored in Stata Version 14. Data were analysed to generate frequencies, percentages or proportions and means. Comparisons between groups was done via logistic regression for the calculation of odds ratios and 95% confidence intervals. The results of analysis were presented in the form of graphs, tables, charts, and means and were included in interim and end of campaign reports.

Quality Assurance. Research assistants were trained on data collection methods and were able to consult field supervisors and the principal investigator on any issue that was not clear to them. For quality assurance, the survey supervisors revisited about 10% of the households, not to collect the data again, but to ensure that they were not skipped by the interviewers for eligible

For peer review only - http://bmjopen.bmj.com/site/about/guidelines.xhtml

respondents. The surveys were conducted about two weeks after completion of the second round of the vaccination campaign to minimize recall bias.

Sub-study 2, staff assessment. Staff were assessed on their level of knowledge on the cause of cholera, the importance of safe water in cholera prevention, the target age group for cholera vaccination, and knowledge about AEFI and the procedures for care should subjects experience an AEFI. Staff were surveyed twice with each survey taking place within two weeks after administration of the first and second OCV rounds, respectively. All staff who participated directly by administering the vaccines or indirectly through supervisory roles and who were present at the workstation during the study period were enrolled in the survey. For the staff survey, structured questions were administered on paper questionnaires that allowed for adding text to explain the answers (open-ended questions). Most of the vaccine staff had taken part in other public health campaigns, but none had participated earlier with a campaign to distribute OCV.

Ethical Considerations. This study was conducted as part of the routine MoH operational research for improvement of health services; however, ethical issues were considered and addressed. The proposal was approved by the Makerere University School of Public Health Institutional Review Board (MaKSPH IRB) (no 610 in 2018) and Uganda National Council of Science and Technology. Written informed consent was obtained from all participants in both sub-studies. Participation in the study was voluntary and respondents were free to opt out at any stage of the interviews.

Confidentiality was observed at all stages of the study. No names or personal identifiers were included on the questionnaires. The research assistants underwent training on interview techniques, neutrality, and research ethics. The benefits of the study to the staff included the ability to express themselves, provide feedback and observations that in turn might lead to improvements in supportive services for their training and work.

Patient and Public Involvement. This research was done without research subject involvement. The time was inadequate to involve the subjects prior to the vaccine campaign. They were also not invited to contribute to the writing or editing of this document for readability or accuracy. However, the findings of the study were disseminated to the Hoima district administration, MoH and other policymakers to use them to strengthen health service interventions and future OCV campaigns.

For peer review only - http://bmjopen.bmj.com/site/about/guidelines.xhtml

RESULTS

Sub-study One, community survey results. The community surveys were carried out in the four sub-counties in Hoima districts of Buseruka, Kabwoya, Kyangwali and Kigorobya as shown in Figure 2 and Table 1. [Insert Figure 2 and Table 1] A total of 209 households, including 1,274 individuals, were surveyed. Most (96%) of the respondents were household heads or their spouses. All respondents confirmed that they were living in the targeted OCV area at the time of the campaign. Fifty-one (51%) of the respondents had primary education, 17% had secondary education, 1% had tertiary education, and the remaining 31% had no education. The respondents were aged 18 – 89 years with a mean age of 40 years. Both sexes were present, with no statistically significant difference.

By verbal reports, 94% (95% CI: 92-95%) of the residents received at least one dose and 78% (95% CI: 76-81%) received two doses of OCV. From verbal reporting, 91% (95% CI: 90-93%) of residents received vaccine during the first round and 81% (95% CI: 78-83%) received vaccine during the second round. For many of the households, a vaccine card was available, and the vaccination card was used to confirm vaccination status. Using information from the card only, coverage was 84% (95% CI: 82-86%) and 65% (95% CI: 62-67%) for round one and round two respectively and the two-dose coverage was 62% (95% CI: 60-65%). Coverage rates are shown on Table 2. [Insert Table 2]

Among those who did not receive a dose of vaccine, over half of these missed doses (254 of the 357 missed doses during the two rounds) were because the person was not at home at the time of vaccination or was out of town. In a few cases, the vaccine team missed the household, accounting for 53 missed doses. Refusing to take vaccine was not reported.

Reported AEFIs. Overall, 71 individuals of 1,274 respondents (5.6%) reported an AEFI (Table 3). Determining a causal relation between the vaccination and the reported symptoms was not attempted. [Insert Table 3]

Most AEFIs were considered mild or moderate, but 8 (0.6%) persons reported an AEFI as severe. Most (60%) of the persons reporting an AEFI did not seek treatment including 60% of those reporting a severe AEFI. 29.6% of the reported adverse events occurred in the first round, 40.9% in the second round and 29.6% in both rounds. The most common symptoms were abdominal pain (15), diarrhoea, (9), fever, nausea, and headache (each 6) reports. Table 4

For peer review only - http://bmjopen.bmj.com/site/about/guidelines.xhtml

provides additional information on the AEFIs. The reported AEFIs were infrequent relative to the number of doses distributed and there were no serious adverse events reported. [Insert Table4]

Community knowledge of oral cholera vaccines.

A majority (77%) of the respondents understood that vaccine was one of the ways to prevent cholera. There was a statistically significant association between education level and knowledge about OCV with those having at least a primary school education being almost twice as likely to know the number of required doses as compared to those with no education (OR 1.90, 95% CI 1.06, 3.44 (P = 0.03).

Sub-study Two, staff survey. A total of 242 and 125 staff responded to the first and second knowledge and practice (KP) surveys (KP1 and KP2). Most respondents were vaccination team members (89% and 87% in vaccine rounds 1 and 2, respectively). Almost all the respondents were knowledgeable about the cause of cholera, the importance of safe water in cholera prevention and the vaccine target group, but were less knowledgeable regarding potential adverse events following administration (AEFI) or how to advise vaccinees, with 29% and 16% being less informed about AEFI during the first and second surveys.

When staff were asked to suggest areas that needed improvement in future OCV campaigns, more than 10% suggested more timely payment of allowances, more time to sensitize and inform the communities on the benefits of the vaccine, and better transportation and facilitation allowances (payments to health workers to cover the cost they incurred when administering the vaccines or conducting activities related to the OCV campaign). Other suggestions included use of both static and mobile vaccination points, provision of gumboots, umbrellas, and more areas for vaccine storage in sub-counties where vaccine would be more accessible, more workers for hard to reach areas, and an increase in the number of vaccine days to complete the vaccinations and increase coverage.

DISCUSSION

The results of this monitoring and evaluation exercise documented important findings on the OCV campaign, the knowledge and practices of both the community and the health staff involved in the campaign and implications for the conduct of future OCV campaigns as part of an integrated cholera control strategy. These findings suggest that the OCV campaign in Hoima successfully provided the vaccine to a very large proportion of the target population in Hoima district, western Uganda. Approximately 93.6% of respondents reported receiving at least one

For peer review only - http://bmjopen.bmj.com/site/about/guidelines.xhtml

dose and 78.3% reported receiving two doses among residents. Given the mobile and transient nature of this population, this was noteworthy, and suggests that even better coverage may be possible for more settled populations in Uganda.

Since this was the first such campaign with OCV, there was concern that the population might be reluctant to accept it. This is a vaccine with which they were not familiar, it was given orally to all ages rather by injection to children, and two doses were required. Despite these potential constraints, we found that most people accepted taking the vaccine readily; however, some were not at home resulting in missed vaccinations.

High vaccination coverage is especially important when one is attempting to achieve herd protection. Since it is estimated that herd protection can be achieved with a coverage even lower than 90%;[19] the high coverage achieved in this campaign would be expected to induce significant indirect protection even among those who did not receive vaccine.[19 20]

It was noted in the administrative report from the MoH and during a stakeholder's meeting that one of the reasons for the reduction in the coverage during the second OCV dose was the unpredictable campaign dates for the second round. The vaccine for the second round had to be shipped and cleared through customs, and the timing for this was not certain. To avoid this problem in the future, a mechanism needs to be established to provide a better timeline for receipt of the vaccine shipments.

Community Reception to OCV. As with previous OCV campaigns outside Uganda, very few AEFI were reported.[21 22] Most of adverse events were considered mild or moderate and were self-limited. Despite the low prevalence of AEFI, the survey exposed the need to better inform the community about seeking treatment for more severe adverse events or for those that do not quickly resolve. This was especially true for families with little education who were less likely to seek medical attention for severe AEFI (data not shown). Notably, members in the community demonstrated good understanding of the rationale for the vaccine; however, a key takeaway from the survey was a need to better communicate the number of required doses, given that those with more education were twice as likely than those with no education to know the number of doses needed.

For peer review only - http://bmjopen.bmj.com/site/about/guidelines.xhtml

Staff Reception to OCV. Inclusion of staff KP survey contributed to the success of the project by identifying gaps among the staff knowledge and performance. Questioning the vaccine staff about their training and their experience in the field is not a common activity when conducting monitoring and evaluation activities during OCV campaigns. Many people had to be mobilized quickly and these were the key people who interacted with the communities. It was important that the staff accurately represent the campaign as an integrated cholera prevention program, but this was the first time these people carried out this role. The MoH felt it important to monitor their knowledge and behaviors as well as any constraints they felt in carrying out their functions. While they were generally knowledgeable about the disease and about the vaccine, these staff needed additional training regarding recognizing and managing AEFIs. They also faced challenges regarding logistical support. After the first staff KP survey, these gaps were communicated to the MoH so that appropriate actions could be taken to ensure that these gaps were addressed prior to the second round.

Most other OCV campaigns have also reported high coverage rates. These have included reports from Bangladesh[23 24], Malawi[25 26], Mozambique[21], Democratic Republic of Congo [27], Zambia [28], South Sudan [29 30], Iraq[31], Haiti[32], and Guinea [33]. Clearly, OCV is well accepted among these very diverse population groups where the vaccine campaigns have been carried out.

Important limitations of this study need to be mentioned. Ideally, one would prefer to conduct community studies prior to a campaign to understand knowledge and attitudes about cholera to improve communications regarding the upcoming campaign as part of an integrated strategy to control cholera. However, since the campaign was carried out on an emergency basis during an outbreak, a study prior to the campaign was not possible. Secondly, a community survey immediately after the first round might have provided feedback to the teams that would have improved the coverage for the second round. It should also be noted that a single informant provided information about receipt of the vaccine for all members of the household, so this informant might have incorrect information concerning one or more members of the household; however, since the vaccine was directly given to the household members together, it seems that inaccuracies would be minimal. The community KP survey did not include questions on attitudes regarding cholera. Since the survey had to be carried out very quickly following the campaign, and since the survey was targeted to identify issues that would be immediately relevant to campaign performance, it was felt that understanding attitudes regarding cholera, even though

For peer review only - http://bmjopen.bmj.com/site/about/guidelines.xhtml

important, would have required other qualitative methods requiring more time than was available. Similarly, direct observation of the training and coordination meetings would have been useful to independently assess the efficiency and effectiveness of these training and coordination meetings. Furthermore, there was no list of all workers in the campaign and many of the workers who participated in the second round had left prior to administering the questionnaire; thus, there were fewer respondents in the second round and the proportion of all workers who participated could not be determined precisely. Finally, it was not possible, given the time constraints, to fully integrate WASH interventions together with the OCV campaign, or to monitor and evaluate community and staff responsiveness to such integration.

In this outbreak 2,122 cholera cases and 44 deaths were reported, nearly all before the OCV campaign and over half occurred in the first two weeks of the outbreak. Of note, the outbreak started in February 2018 at about the same time the application for preventive use of OCV was being was submitted. The original application proposed a series of preventive campaigns over the next year, and Hoima, as well as neighbouring districts in western Uganda, were targeted for vaccination in the first round of these preventive campaigns. However, when the outbreak was identified, plans were quickly shifted so that an emergency campaign could be implemented to control the outbreak. Even though this emergency response was planned as quickly as possible, in fact, the outbreak was essentially over before the vaccine campaign could start, so it had no impact on the outbreak itself, but likely reduced the risk for future outbreaks.

Though the outbreak started with the influx of the refugees from DRC into Uganda, it quickly spread to the refugee host communities in Hoima. Therefore, to prevent rapid spread, improvement of cholera prevention measures for both the refugees and the host communities is paramount during resettlement.

CONCLUSION

This study suggests that the OCV campaign in Hoima district to prevent cholera was successful and achieved a high level of coverage in this population at high risk. However, there was need to devote more effort on community sensitisation on the benefits of vaccination, as well as improving some logistic support during the campaign.

For peer review only - http://bmjopen.bmj.com/site/about/guidelines.xhtml

While a rapid response to this outbreak was appropriate, in fact, even with a rapidly organized campaign, the outbreak was over before the vaccine could be given; thus, the vaccine had no impact on this outbreak. Nevertheless, this area had already been identified as a hotspot, and it would have been targeted if the planned preventive campaign had proceeded as originally planned. Planners must realize that an area identified as a hotspot might experience an outbreak while preparations are underway for a preventive campaign and take this into account when allocating vaccine for preventive vs emergency campaigns. Since these are areas where cholera risk is high, outbreaks are likely to occur in these areas if there are delays in implementing preventive campaigns.

For peer review only

FIGURE LEGENDS

Figure 1. Epicurve of the Hoima Outbreak, 2018 with events identified in response to the outbreak

Figure 2: Map of Hoima District, showing sub-counties that received OCV and households where interviews were conducted (red dots)

For peer review only

For peer review only - http://bmjopen.bmj.com/site/about/guidelines.xhtml

Table 1. Age and Sex Breakdown of the Participants in the Cluster Survey

Age Group (years)	Male	Female	Total
**1 to 4**	98	93	191
**5 to 14**	236	207	443
**15 to 44**	210	285	495
**45+**	80	65	145
**Total**	625	650	1274

Table 2: Vaccination coverage post OCV campaign, Hoima District, Uganda, 2018.

Total surveyed = 1274	Round 1	Round 2	Received only one dose	Received two doses	Received at least one dose
**Reported number (%) (95% CI)**	1,164 (91.4) (89.7-92.8)	1,027 (80.6) (78.3-82.7)	195 (15.3) (13.4-17.4)	998 (78.3) (76.0-80.5)	1193 (93.6) (92.2-94.9)
**Confirmed by availability of the vaccination card (%) (95% CI)**	1,065 (83.6) (81.5-85.5)	823 (64.6) (61.9-67.2)	142 (11.1) (9.5-13.0)	792 (62.2) (59.5-64.8)	934 (73.3) (70.8-75.7)

Table 3: Treatment and Resolution of Adverse Events Following Immunization in Hoima District, Uganda, 2018.

Symptoms	Treatment		Status		
	**No treatment (%)**	**Treated (%)**	**Recovered (%)**	**Ongoing (%)**	**Improved, not to baseline (%)**
**Mild**	24(80.0)	6(20.0)	29(96.7)	1(3.3)	0(0.0)
**Moderate**	11(39.3)	17(60.7)	23(82.1)	3(10.7)	2(7.1)
**Severe**	8(61.5)	5(38.5)	10(76.9)	0(0.0)	3(23.1)

For peer review only

Table 4. Onset and frequency of symptoms reported as adverse events	<6 hours	6-12 hours	12-24 hours	1-7 days	8-14 days	Total
**Diarrhoea**	3	2	2	2	0	9
**Vomiting**	3	0	0	2	0	5
**Nausea**	6	0	0	0	0	6
**Abdominal pain**	15*	0	0	0	0	15
**Stomach gurgling**	0	3	0	1	0	4
**Mouth ulcers**	0	0	0	0	1	1
**Cough**	1	1	0	3	1	6
**Felt feverish**	1	1	0	6	1	9
**Poor appetite**	1	0	0	0	0	1
**Dizziness**	0	3	0	0	0	3
**Fainted**	0	1	0	0	0	1
**Itching**	0	0	0	0	1	1
**Weakness**	0	0	0	1	0	1
**Headache**	3	0	2	1	0	6
**Other**	1	0	0	2	0	3
**Total**	34	11	4	18	4	71

- **Three persons reported abdominal pain in one household**

For peer review only - http://bmjopen.bmj.com/site/about/guidelines.xhtml

Acknowledgements

The authors thank the persons and institutions who supported Hoima district OCV monitoring and evaluation study. Special thanks are extended to the research assistants who dedicated their time to meticulously collect the data and the persons who collaborated in the preparation of the different key documents used in this study. We thank the staff, the communities in the study area and the local leadership of Hoima districts for the cooperation during this study. We are grateful to Ambrose Buyinza Wabwire for technical support with drawing of the maps.

We thank the Ministry of Health leadership for guidance; the Office of the Prime Minister (OPM) for the permission to access data on the refugee population. We are grateful to the following institutions and bodies; Ministry of Health, Makerere University School of Public Health, World Health Organisation, UNICEF and UNHCR for technical guidance.

Footnotes

Contributors: Conceived and designed the study: GB, MR, WAB, ABS, CGO, DAS. Discussed, critically revised and approved the study protocol: GB, MR, WAB, CGO, DAS. Performed the research: GB, MR, WAB, AO, FR, IA. Analysed the data: GB, MR, WAB, AO, DAS. Wrote the first draft: GB, MR. Wrote the final DAS. Elaborated, discussed and approved the final version: GB, MR, AB, ABS, AO, FR, IA, CGO, and DAS.

Funding: Funding for the study was provided by the Bill and Melinda Gates Foundation (OPP1148763) which provided financial support through the John Hopkins University, Delivery of Oral Cholera Vaccine Effectively (DOVE) project. The funding agency had no role in collecting, analysing or interpreting the results.

Declaration of the conflict of interest

The authors have no conflict of interest to declare.

Additional data sharing: Data are available upon reasonable request

For peer review only

REFERENCES

1. Clemens JD, Nair GB, Ahmed T, et al. Cholera. *Lancet* 2017 doi: 10.1016/S0140-6736(17)30559-7
2. Cholera vaccines: WHO position paper - August 2017. *Wkly Epidemiol Rec* 2017;92(34):477-98.
3. Kirigia JM, Sambo LG, Yokouide A, et al. Economic burden of cholera in the WHO African region. *BMC Int Health Hum Rights* 2009;9:8. doi: 10.1186/1472-698X-9-8
4. Lessler J, Moore SM, Luquero FJ, et al. Mapping the burden of cholera in sub-Saharan Africa and implications for control: an analysis of data across geographical scales. *Lancet* 2018;391(10133):1908-15. doi: 10.1016/S0140-6736(17)33050-7
5. Bwire G, Ali M, Sack DA, et al. Identifying cholera "hotspots" in Uganda: An analysis of cholera surveillance data from 2011 to 2016. *PLoS Negl Trop Dis* 2017;11(12):e0006118. doi: 10.1371/journal.pntd.0006118
6. Cummings MJ, Wamala JF, Eyura M, et al. A cholera outbreak among semi-nomadic pastoralists in northeastern Uganda: epidemiology and interventions. *Epidemiol Infect* 2012;140(8):1376-85. doi: 10.1017/S0950268811001956 [published Online First: 2011/09/29]
7. Bwire G, Mwesawina M, Baluku Y, et al. Cross-Border Cholera Outbreaks in Sub-Saharan Africa, the Mystery behind the Silent Illness: What Needs to Be Done? *PLoS One* 2016;11(6):e0156674. doi: 10.1371/journal.pone.0156674
8. Bwire G, Malimbo M, Maskery B, et al. The burden of cholera in Uganda. *PLoS Negl Trop Dis* 2013;7(12):e2545. doi: 10.1371/journal.pntd.0002545
9. Oguttu DW, Okullo A, Bwire G, et al. Cholera outbreak caused by drinking lake water contaminated with human faeces in Kaiso Village, Hoima District, Western Uganda, October 2015. *Infect Dis Poverty* 2017;6(1):146. doi: 10.1186/s40249-017-0359-2
10. Alajo SO, Nakavuma J, Erume J. Cholera in endemic districts in Uganda during El Nino rains: 2002-2003. *Afr Health Sci* 2006;6(2):93-7. doi: 10.5555/afhs.2006.6.2.93 [published Online First: 2006/08/19]
11. Montgomery M, Jones MW, Kabole I, et al. No end to cholera without basic water, sanitation and hygiene. *Bull World Health Organ* 2018;96(6):371-71A. doi: 10.2471/BLT.18.213678
12. Legros D, Partners of the Global Task Force on Cholera C. Global Cholera Epidemiology: Opportunities to Reduce the Burden of Cholera by 2030. *J Infect Dis* 2018 doi: 10.1093/infdis/jiy486
13. Deployments from the oral cholera vaccine stockpile, 2013-2017. *Wkly Epidemiol Rec* 2017;92(32):437-42.
14. Odevall L, Hong D, Digilio L, et al. The Euvichol story - Development and licensure of a safe, effective and affordable oral cholera vaccine through global public private partnerships. *Vaccine* 2018;36(45):6606-14. doi: 10.1016/j.vaccine.2018.09.026
15. Bwire G, Munier A, Ouedraogo I, et al. Epidemiology of cholera outbreaks and socio-economic characteristics of the communities in the fishing villages of Uganda: 2011-2015. *PLoS Negl Trop Dis* 2017;11(3):e0005407. doi: 10.1371/journal.pntd.0005407

For peer review only - http://bmjopen.bmj.com/site/about/guidelines.xhtml

16. Uganda_Bureau_of_Statistics_(UBOS). Demographic and Health Survey. 2016 Kampala and Rockville Maryland2016 [Available from: https://www.dhsprogram.com/pubs/pdf/FR333/FR333.pdf accessed Sept 18. 2020 2020.
17. Henderson RH, Sundaresan T. Cluster sampling to assess immunization coverage: a review of experience with a simplified sampling method. *Bull World Health Organ* 1982;60(2):253-60.
18. Food_and_Drug_Administration. Guidance for Industry. Toxicity Grading Scale for Healthy Adult and Adolescent Volunteers Enrolled in Preventive Vaccine Clinical Trials Rockville, Maryland USA: Food and Drug Administration, Center for Biologics Evaluation and Research 2007 [cited 2020. Available from: https://www.fda.gov/media/73679/download accessed September 22 2020.
19. Longini IM, Jr., Nizam A, Ali M, et al. Controlling endemic cholera with oral vaccines. *PLoS Med* 2007;4(11):e336. doi: 10.1371/journal.pmed.0040336 [published Online First: 2007/11/30]
20. Ali M, Emch M, von Seidlein L, et al. Herd immunity conferred by killed oral cholera vaccines in Bangladesh: a reanalysis. *Lancet* 2005;366(9479):44-9.
21. Sema Baltazar C, Rafael F, Langa JPM, et al. Oral cholera vaccine coverage during a preventive door-to-door mass vaccination campaign in Nampula, Mozambique. *PLoS One* 2018;13(10):e0198592. doi: 10.1371/journal.pone.0198592
22. Phares CR, Date K, Travers P, et al. Mass vaccination with a two-dose oral cholera vaccine in a long-standing refugee camp, Thailand. *Vaccine* 2016;34(1):128-33. doi: 10.1016/j.vaccine.2015.10.112
23. Khan AI, Khan IA, Siddique SA, et al. Feasibility, coverage and cost of oral cholera vaccination conducted by icddr,b using the existing national immunization service delivery mechanism in rural setting Keraniganj, Bangladesh. *Hum Vaccin Immunother* 2019;15(6):1302-09. doi: 10.1080/21645515.2018.1528833
24. Uddin MJ, Wahed T, Saha NC, et al. Coverage and acceptability of cholera vaccine among high-risk population of urban Dhaka, Bangladesh. *Vaccine* 2014;32(43):5690-5. doi: 10.1016/j.vaccine.2014.08.021
25. Grandesso F, Rafael F, Chipeta S, et al. Oral cholera vaccination in hard-to-reach communities, Lake Chilwa, Malawi. *Bull World Health Organ* 2018;96(12):817-25. doi: 10.2471/BLT.17.206417
26. Sauvageot D, Saussier C, Gobeze A, et al. Oral cholera vaccine coverage in hard-to-reach fishermen communities after two mass Campaigns, Malawi, 2016. *Vaccine* 2017;35(38):5194-200. doi: 10.1016/j.vaccine.2017.07.104
27. Massing LA, Aboubakar S, Blake A, et al. Highly targeted cholera vaccination campaigns in urban setting are feasible: The experience in Kalemie, Democratic Republic of Congo. *PLoS Negl Trop Dis* 2018;12(5):e0006369. doi: 10.1371/journal.pntd.0006369
28. Poncin M, Zulu G, Voute C, et al. Implementation research: reactive mass vaccination with single-dose oral cholera vaccine, Zambia. *Bull World Health Organ* 2018;96(2):86-93. doi: 10.2471/BLT.16.189241

For peer review only - http://bmjopen.bmj.com/site/about/guidelines.xhtml

29. Parker LA, Rumunu J, Jamet C, et al. Neighborhood-targeted and case-triggered use of a single dose of oral cholera vaccine in an urban setting: Feasibility and vaccine coverage. *PLoS Negl Trop Dis* 2017;11(6):e0005652. doi: 10.1371/journal.pntd.0005652
30. Porta MI, Lenglet A, de Weerdt S, et al. Feasibility of a preventive mass vaccination campaign with two doses of oral cholera vaccine during a humanitarian emergency in South Sudan. *Trans R Soc Trop Med Hyg* 2014;108(12):810-5. doi: 10.1093/trstmh/tru153
31. Lam E, Al-Tamimi W, Russell SP, et al. Oral Cholera Vaccine Coverage during an Outbreak and Humanitarian Crisis, Iraq, 2015. *Emerg Infect Dis* 2017;23(1):38-45. doi: 10.3201/eid2301.160881
32. Tohme RA, Francois J, Wannemuehler K, et al. Oral Cholera Vaccine Coverage, Barriers to Vaccination, and Adverse Events following Vaccination, Haiti, 2013. *Emerg Infect Dis* 2015;21(6):984-91. doi: 10.3201/eid2106.141797
33. Luquero FJ, Grout L, Ciglenecki I, et al. First outbreak response using an oral cholera vaccine in Africa: vaccine coverage, acceptability and surveillance of adverse events, Guinea, 2012. *PLoS Negl Trop Dis* 2013;7(10):e2465. doi: 10.1371/journal.pntd.0002465

For peer review only

For peer review only - http://bmjopen.bmj.com/site/about/guidelines.xhtml

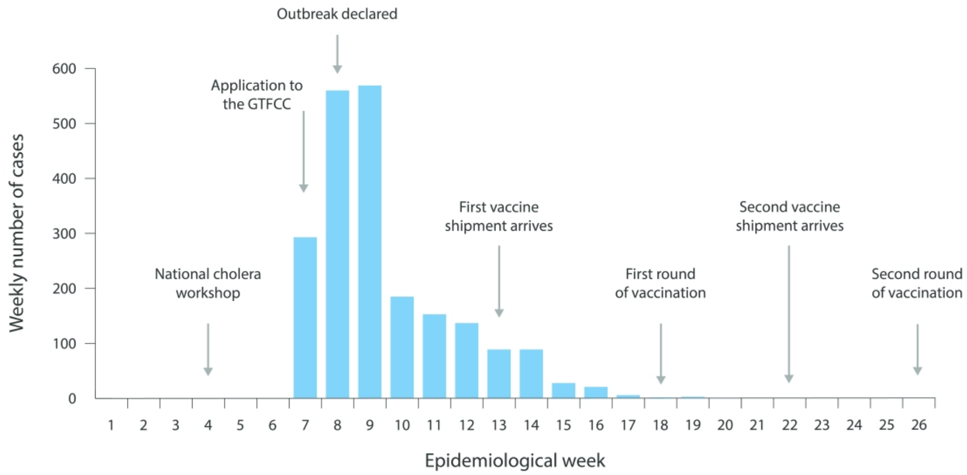

Figure 1. Epicurve of the Hoima Outbreak, 2018 with events identified in response to the outbreak

542x305mm (300 x 300 DPI)

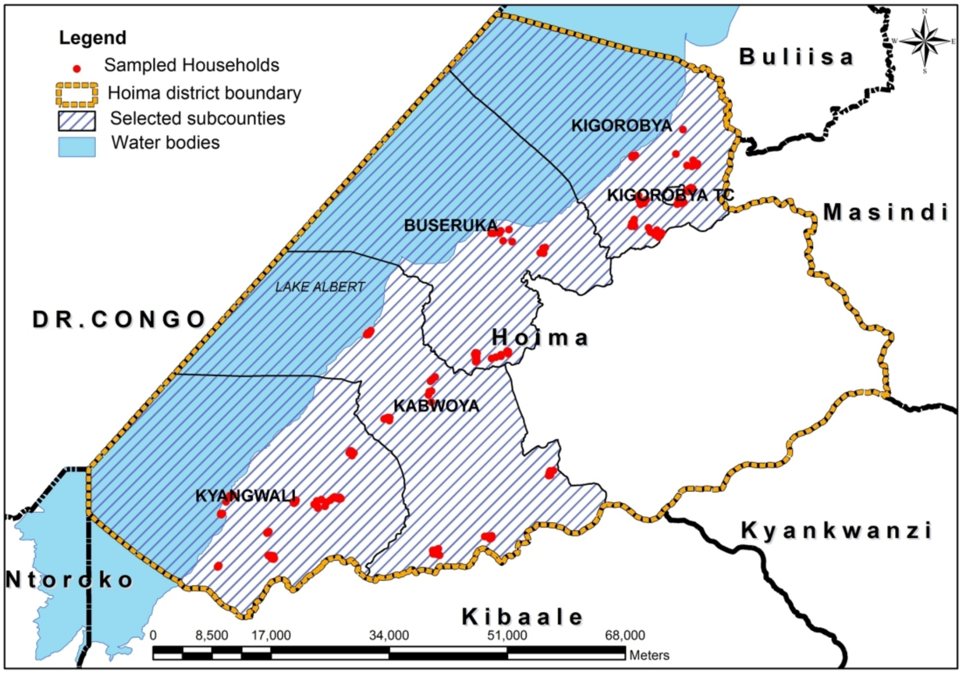

Figure 2: Map of Hoima District, showing sub-counties that received OCV and households where interviews were conducted (red dots)

170x119mm (300 x 300 DPI)

BMJ Open

Use of surveys to evaluate an integrated oral cholera vaccine campaign in response to a cholera outbreak in Hoima District, Uganda

Journal:	*BMJ Open*
Manuscript ID	bmjopen-2020-038464.R3
Article Type:	Original research
Date Submitted by the Author:	17-Nov-2020

Complete List of Authors:	Bwire, Godfrey; Republic of Uganda Ministry of Health, Community Health<br>Roskosky, Mellisa; Johns Hopkins University Bloomberg School of Public Health, International Health<br>Ballard, Anne; Johns Hopkins University Bloomberg School of Public Health, Center for Communications Programs<br>Brooks, W; Johns Hopkins University Bloomberg School of Public Health, International Health<br>Alfred, Okello; Makerere University College of Health Sciences<br>Rafael, Florentina; World Health Organization, Infectious Hazard Management<br>Ampeire, Immaculate; Republic of Uganda Ministry of Health, Expanded Programme on Immunization<br>Orach, Christopher; Makerere University College of Health Sciences<br>Sack, David; Johns Hopkins University Bloomberg School of Public Health, International Health

**Primary Subject Heading**:	Global health
Secondary Subject Heading:	Epidemiology, Health services research, Infectious diseases, Public health
Keywords:	Gastrointestinal infections < GASTROENTEROLOGY, International health services < HEALTH SERVICES ADMINISTRATION & MANAGEMENT, Epidemiology < INFECTIOUS DISEASES, Infection control < INFECTIOUS DISEASES, Public health < INFECTIOUS DISEASES, Tropical medicine < INFECTIOUS DISEASES

SCHOLARONE™
Manuscripts

I, the Submitting Author has the right to grant and does grant on behalf of all authors of the Work (as defined in the below author licence), an exclusive licence and/or a non-exclusive licence for contributions from authors who are: i) UK Crown employees; ii) where BMJ has agreed a CC-BY licence shall apply, and/or iii) in accordance with the terms applicable for US Federal Government officers or employees acting as part of their official duties; on a worldwide, perpetual, irrevocable, royalty-free basis to BMJ Publishing Group Ltd ("BMJ") its licensees and where the relevant Journal is co-owned by BMJ to the co-owners of the Journal, to publish the Work in this journal and any other BMJ products and to exploit all rights, as set out in our licence.

The Submitting Author accepts and understands that any supply made under these terms is made by BMJ to the Submitting Author unless you are acting as an employee on behalf of your employer or a postgraduate student of an affiliated institution which is paying any applicable article publishing charge ("APC") for Open Access articles. Where the Submitting Author wishes to make the Work available on an Open Access basis (and intends to pay the relevant APC), the terms of reuse of such Open Access shall be governed by a Creative Commons licence – details of these licences and which Creative Commons licence will apply to this Work are set out in our licence referred to above.

Other than as permitted in any relevant BMJ Author's Self Archiving Policies, I confirm this Work has not been accepted for publication elsewhere, is not being considered for publication elsewhere and does not duplicate material already published. I confirm all authors consent to publication of this Work and authorise the granting of this licence.

For peer review only

Use of surveys to evaluate an integrated oral cholera vaccine campaign in response to a cholera outbreak in Hoima District, Uganda

Godfrey Bwire[1,] Mellisa Roskosky[2], Anne Ballard Sara[2], W. Abdullah Brooks[2], Alfred Okello[3], Florentina Rafael [4], Immaculate Ampeire[5], Christopher Garimoi Orach[3], David A. Sack[2].

Authors' affiliations:

1. Ministry of Health, Department of Community Health, Kampala Uganda.
2. Johns Hopkins Bloomberg School of Public Health, Department of International Health, Baltimore, Maryland USA.
3. Makerere University, College of Health Sciences, Kampala, Uganda.
4. World Health Organization, Department of Infectious Hazard Management, Geneva, Switzerland.
5. Ministry of Health, Uganda National Expanded Program on Immunization

*Corresponding author.

Professor David A. Sack, M, D.
Johns Hopkins Bloomberg School of Public Health
615 North Wolfe Street
Baltimore, Maryland 21205 USA
Phone +1 443 287 8795
Email: dsack1@jhu.edu

Number of words (Excluding abstract): 4,735
Number of words in abstract: 264
Number of tables: 4
Number of figures: 2
Number of references: 33
Number of supplemental materials: 0

Key words: cholera, vaccination, Uganda, monitoring, coverage, outbreak, sub-Saharan Africa, oral cholera vaccine, Vaccine campaign

For peer review only - http://bmjopen.bmj.com/site/about/guidelines.xhtml

ABSTRACT

Objectives To evaluate the quality and coverage of the campaign to distribute oral cholera vaccine during a cholera outbreak in Hoima, Uganda to guide future campaigns of cholera vaccine.

Design Survey of communities targeted for vaccination to determine vaccine coverage rates and perceptions of the vaccination campaign, and a separate survey of vaccine staff who carried out the campaign.

Setting Hoima District, Uganda.

Participants Representative clusters of households residing in the communities targeted for vaccination and staff members who conducted the vaccine campaign.

Results Among 209 households (1,274 individuals) included in the coverage survey, 1193 (94%; 95% CI: 92-95%) reported receiving at least one OCV dose and 998 (78%; 95% CI: 76-81%) reported receiving two doses. Among vaccinated individuals, minor complaints were reported by 71 persons (5.6%). Individuals with 'some' education (primary school or above) were more knowledgeable regarding the required OCV doses compared to non-educated (p value = 0.03). Factors negatively associated with campaign implementation included community sensitisation time, staff payment, and problems with field transport. Although the campaign was carried out quickly, the outbreak was over before the campaign started. Most staff involved in the campaign (93%) were knowledgeable about cholera control; however, 29% did not clearly understand how to detect and manage adverse events following immunization.

Conclusion The campaign achieved high OCV coverage, but the surveys provided insights for improvement. To achieve high vaccine coverage, more effort is needed for community sensitisation, and additional resources for staff transportation and timely payment for campaign staff is required. Pre and post-test assessment of staff training can identify and address knowledge and skill gaps.

For peer review only - http://bmjopen.bmj.com/site/about/guidelines.xhtml

Strengths and limitations of this study

- The cluster survey of households in communities targeted for vaccination efficiently documented actual vaccine coverage in the target population.
- The cluster surveys of households identified mild adverse events not identified during the campaign and identified the emphasize the second dose, especially among less educated groups.
- Surveys of the vaccination staff immediately following each round identified certain weaknesses in staff orientation as well as constraints to their job performance in the field.
- The household surveys obtained data from a single spokesperson for the household rather than from each individual which might have introduced some uncertainty in the household data.
- Evaluation of the vaccination staff was carried through surveys and would have benefited by direct observation of the training and the field performance.

For peer review only - http://bmjopen.bmj.com/site/about/guidelines.xhtml

INTRODUCTION

Cholera, a preventable and treatable disease is characterized by profuse watery diarrhoea caused by infection of the intestine with the bacterium *Vibrio cholerae.*[1] Cholera is a major cause of morbidity and mortality in several countries in sub-Saharan Africa where cholera outbreaks also negatively affect development due to associated high economic burden. [2 3] Between 2010 and 2016 an average 141,918 incident cases annually were reported from sub-Saharan African countries, including Uganda.[4] In Uganda, cholera outbreaks occurred as both endemic and epidemic disease. Epidemic disease occurred in northern and eastern Uganda districts [5] and are thought to be worsened by contamination of water due to poor sanitation.[6] Cholera outbreaks especially occur in districts along the international borders with the Democratic Republic of the Congo (DR Congo), South Sudan and Kenya and along the Great Lakes.[5 7] These districts include Hoima, where cholera is endemic. [5 8-10]

There has been debate in the public health community on best practices for endemic and epidemic cholera disease control, with some preferring to focus on WASH interventions, and others advocating for OCV for both endemic and epidemic disease control.[11]. In part, this has been facilitated by a relative lack of experience with OCV and concern that excess reliance on vaccine might negatively affect essential infrastructural development and hygienic practices. The World Health Organization (WHO) recommends an integrated approach to cholera prevention where water, sanitation and hygiene (WaSH) interventions are complemented by vaccine campaigns which provide oral cholera vaccine (OCV) to persons living in areas considered high risk.[2 12] These vaccine campaigns may be either preventive, in which the vaccine is targeted to cholera hotspots, or reactive in which the campaign is implemented in response to an outbreak or a humanitarian emergency.[13]

Two WHO-prequalified currently OCVs are available from the global stockpile: Shanchol (Shantha Biotechnics Limited, India) and Euvichol (Eubiologics Co., Ltd., Korea).[2] The standard immunization schedule consists of two doses given at an interval of at least two weeks to all persons in the target area above one year of age. While there is increasing use of OCV to control outbreaks, preventive use is constrained due to inadequate vaccine supply.[14] Since creation of a global OCV stockpile in July, 2013, several OCV campaigns had been successfully implemented[13 14] but it is still important to document national campaign experiences as well as monitoring and evaluation activities, to continually improve the effectiveness and efficiency of vaccine campaigns.

For peer review only - http://bmjopen.bmj.com/site/about/guidelines.xhtml

The Ugandan Ministry of Health (MoH) had prepared plans for OCV campaigns in the areas identified as cholera hotspots starting in the western districts of Uganda (including Hoima), near the border with DR Congo and close to, or adjoining Lake Albert. These hotspot districts and their specified sub-counties were confirmed during a national cholera workshop in Kampala on 29-31 January 2018. This workshop led to the development of an application for OCV to the Global Taskforce for Cholera Control (GTFCC) which was submitted on 14 February 2018. The application proposed providing OCV in these identified hotspots as a preventive strategy. However, while preparations for these campaigns were underway, an outbreak was declared in Hoima district on 23 February 2018. The earliest cases were identified among DR Congo refugees, but then other cases were seen among the non-refugee Ugandan population. The MoH responded to the outbreak with multisectoral interventions, including proper case management, promotion of access to safe water and improved sanitation (WaSH), enhanced cholera surveillance, as well as infection control and health education. These measures were then supplemented with plans for an emergency OCV campaign. Thus, the original plans for a preventive OCV campaign were shifted to an emergency response to control the outbreak. The first doses of vaccine arrived on 28 March and the first round of vaccinations started on 2 May. The doses for the second round arrived on 29 May and the second round started on 26 June. A door-to-door strategy was used to deliver two doses of vaccine to an estimated 360,000 people, including pregnant women, over the age of one year residing in the four targeted sub-counties. To carry out the campaign, the MoH organized all activities including logistics, community mobilisation and implementation, coordinating ground activities through an assigned point person. Many stakeholders contributed to the campaign including the Hoima district local government, WHO, UNICEF, UNHCR and Médecins sans Frontiers (MSF). Prior to the campaign, the stakeholders met to define and coordinate their complementary tasks.

The epidemic curve based on a line list of cases and deaths by date and stated nationality is shown on Figure 1. Over the course of the outbreak, 2,122 cases with 44 deaths (case fatality rate, 2.1%) were reported. Sixty six percent (1,410) of the cases and 64% (28) of the deaths occurred during the first two weeks of the outbreak. Many of the cases and deaths (1276 and 32, or 60% and 73%, respectively) occurred among persons who were from DR Congo, and the refugees developed cholera symptoms soon after arrival in Uganda. Among the 44 deaths reported, 25 (57%) occurred in the community, not in the health facility. Nineteen of the fatal cases were treated at the health facility; the case fatality ratio (CFR) for facility-treated patients was 0.9%. Although the emergency vaccination campaign intended to control the outbreak,

For peer review only - http://bmjopen.bmj.com/site/about/guidelines.xhtml

because the outbreak was so sudden and so short-lived, the campaign could only be initiated after the outbreak had already declined.

Rationale. While vaccines are commonly used in Uganda, especially through the longstanding EPI program (Expanded Programme on Immunization), this was the first OCV use in Uganda, and there was no prior experience to guide responders and implementers. Thus, this study was carried out with the aim to document campaign activities and to monitor and evaluate its procedures and outcomes that could guide future OCV campaigns. The issues addressed during this study included the knowledge and practices of the campaign staff, vaccine coverage in the targeted areas, and the knowledge and practices of the community. After this initial campaign, the MoH continued its plans for preventive campaigns in the remaining cholera hotspot districts, informed by the lessons learned from this initial emergency use campaign. In an effort to document the impact of OCV on an outbreak in a setting with endemic disease, we undertook this monitoring and evaluation exercise, as described below.

MATERIALS AND METHODS

Study setting. Hoima district is located in western Uganda, across Lake Albert from DR Congo. It has a total area of 5735.5 square kilometres and a projected population of 630,000 persons (2018). The district consists of 13 administrative units as follows: 10 sub-counties (Kyabigambire, Buhimba, Kyangwali, Kabwoya, Bugambe, Kiziranfumbi, Kitoba, Kigorobya, Buseruka and Buhanika), a municipality (Hoima municipality) and two town councils (Kigorobya and Buhima town councils). The major economic activities of the population in Hoima are subsistence agriculture and fishing. Cholera is endemic in the district but the endemicity is localised in some specific sub-counties particularly those with fishing communities.[15] The sub-counties targeted for OCV included Buseruka, Kabwoya, Kangwali, and Kigorobya which together constitute the six administrative units of Kyangwali, Kigorobya, Kabwoya Buseruka, Kigorobya town council and Kyangwali refugee settlement (Old and New) as shown in Figure 2.

Population and design for the Monitoring and Evaluation of the OCV Campaign. Two sub-studies were conducted to assess different aspects of the campaign. In sub-study one, a representative sample of the population that was targeted for vaccination was questioned to determine vaccine coverage rates, detect adverse events following immunization (AEFI) and collect additional information from the communities about the vaccine campaign. Sub-study two consisted of a survey among the campaign staff who participated in the OCV campaign after each round to assess their knowledge and practices.

For peer review only

For peer review only - http://bmjopen.bmj.com/site/about/guidelines.xhtml

Sub-study 1, community assessment. Sub-study one was a two-stage, cluster survey conducted in the vaccine target area, consisting of 31 clusters, each cluster consisting of 4 to 7 households per cluster. The study population included each person > one year of age who was living in the OCV targeted area at the time of the vaccination campaign. We assumed a household size of five persons based on estimates from a Demographic Health Survey conducted in 2016.[16] The sample was increased in order to raise the analytical power and precision of the surveys and to allow for separate analysis by gender. The formula used for determining sample size was, $n = (z^2pq)/d^2$,[17] where 'n' is the number of people desired for the survey, 'd' is the precision of the result, 'z' is the confidence limit, and 'p' and 'q' correspond to the proportion of persons in the population who are immunised and not immunised, respectively. We chose to use a low coverage of 50%.

To identify the clusters, a list of villages was obtained from each of the four sub-counties targeted for vaccination. From these lists, the Excel random number generator (=RANDBETWEENBOTTOM, TOP) was used to select the 31 villages from which households were selected. The number of villages per sub-county was proportionate to the population of the sub-county. The sub-county populations were obtained from the district planning unit. From each selected village a list of households was obtained from the village administrative leader (Local Council (LC) – 1. This is the smallest recognised administrative unit in Uganda. It is headed an elected leader called LC-1) who provided a list of households from which we randomly selected households to interview.

For the household interviews, data were collected through standardized questionnaires during face-to-face interviews conducted by trained research assistants using the local language. Within a selected household the questionnaires were administered to the key respondents (head of the HHs), who represented the entire household and provided information about each member of the household. If a suitable key respondent was absent, additional visits were scheduled. In two households, a person could not be located, and the household was dropped. For each vaccinated person, the research assistants assessed cholera immunization status. Vaccination status was ascertained in two ways: either the informant verbally indicated that the individual had received the dose of vaccine or the vaccination was recorded on the vaccine card. If the informant did not have a vaccine card, the reliability of the vaccination information was validated by asking about the details of the procedures of

For peer review only

the vaccination (e.g. being given recently by mouth to all persons > 1 year of age). None of the residents who were approached refused to answer the survey. The age and sex of the participants in the survey is shown in Table 1.

Adverse Events Following Immunization (AEFI). The occurrence of AEFIs were assessed by asking for symptoms among the vaccine recipients following vaccination. As part of the campaign itself, a routine system for AEFI detection was established in which the vaccine team members advised vaccinees to report to a health worker or to seek care at a health facility if they experienced symptoms following immunization. By contrast, the AEFI surveillance in this sub-study asked the participants who participated in the cluster survey about symptoms they may have experienced. This AEFI sub-study was thus, designed to enhance our understanding of potential AEFIs which may not have been reported through the routine AEFI surveillance.

AEFIs were categorised for each individual member of the household who received a dose of OCV as follows. They were considered mild if the symptoms did not interfere with normal activities; moderate if they interfered somewhat; and severe if the symptoms prevented the individual from continuing normal activities.[18] Persons who reported to be having ongoing symptoms > 72 hours were advised to visit the nearest health facility for more care. Among those reporting symptoms, information was recorded as to whether the person took any medicine or received any treatment to lessen the symptoms.

Data collection and analysis. Data from the community surveys were collected by tablet computers using *Kobo Collect* (https://www.kobotoolbox.org/) installed to record the responses in the field. Data were cleaned, coded, and stored in Stata Version 14. Data were analysed to generate frequencies, percentages or proportions and means. Comparisons between groups was done via logistic regression for the calculation of odds ratios and 95% confidence intervals. The results of analysis were presented in the form of graphs, tables, charts, and means and were included in interim and end of campaign reports.

Quality Assurance. Research assistants were trained on data collection methods and were able to consult field supervisors and the principal investigator on any issue that was not clear to them. For quality assurance, the survey supervisors revisited about 10% of the households, not to collect the data again, but to ensure that they were not skipped by the interviewers for eligible

For peer review only - http://bmjopen.bmj.com/site/about/guidelines.xhtml

respondents. The surveys were conducted about two weeks after completion of the second round of the vaccination campaign to minimize recall bias.

Sub-study 2, staff assessment. Staff were assessed on their level of knowledge on the cause of cholera, the importance of safe water in cholera prevention, the target age group for cholera vaccination, and knowledge about AEFI and the procedures for care should subjects experience an AEFI. Staff were surveyed twice with each survey taking place within two weeks after administration of the first and second OCV rounds, respectively. All staff who participated directly by administering the vaccines or indirectly through supervisory roles and who were present at the workstation during the study period were enrolled in the survey. For the staff survey, structured questions were administered on paper questionnaires that allowed for adding text to explain the answers (open-ended questions). Most of the vaccine staff had taken part in other public health campaigns, but none had participated earlier with a campaign to distribute OCV.

Ethical Considerations. This study was conducted as part of the routine MoH operational research for improvement of health services; however, ethical issues were considered and addressed. The proposal was approved by the Makerere University School of Public Health Institutional Review Board (MaKSPH IRB) (no 610 in 2018) and Uganda National Council of Science and Technology. Written informed consent was obtained from all participants in both sub-studies. Participation in the study was voluntary and respondents were free to opt out at any stage of the interviews.

Confidentiality was observed at all stages of the study. No names or personal identifiers were included on the questionnaires. The research assistants underwent training on interview techniques, neutrality, and research ethics. The benefits of the study to the staff included the ability to express themselves, provide feedback and observations that in turn might lead to improvements in supportive services for their training and work.

Patient and Public Involvement. This research was done without research subject involvement. The time was inadequate to involve the subjects prior to the vaccine campaign. They were also not invited to contribute to the writing or editing of this document for readability or accuracy. However, the findings of the study were disseminated to the Hoima district administration, MoH and other policymakers to use them to strengthen health service interventions and future OCV campaigns.

For peer review only - http://bmjopen.bmj.com/site/about/guidelines.xhtml

RESULTS

Sub-study One, community survey results. The community surveys were carried out in the four sub-counties in Hoima districts of Buseruka, Kabwoya, Kyangwali and Kigorobya as shown in Figure 2 and Table 1. [Insert Figure 2 and Table 1] A total of 209 households, including 1,274 individuals, were surveyed. Most (96%) of the respondents were household heads or their spouses. All respondents confirmed that they were living in the targeted OCV area at the time of the campaign. Fifty-one (51%) of the respondents had primary education, 17% had secondary education, 1% had tertiary education, and the remaining 31% had no education. The respondents were aged 18 – 89 years with a mean age of 40 years. Both sexes were present, with no statistically significant difference.

By verbal reports, 94% (95% CI: 92-95%) of the residents received at least one dose and 78% (95% CI: 76-81%) received two doses of OCV. From verbal reporting, 91% (95% CI: 90-93%) of residents received vaccine during the first round and 81% (95% CI: 78-83%) received vaccine during the second round. For many of the households, a vaccine card was available, and the vaccination card was used to confirm vaccination status. Using information from the card only, coverage was 84% (95% CI: 82-86%) and 65% (95% CI: 62-67%) for round one and round two respectively and the two-dose coverage was 62% (95% CI: 60-65%). Coverage rates are shown on Table 2. [Insert Table 2]

Among those who did not receive a dose of vaccine, over half of these missed doses (254 of the 357 missed doses during the two rounds) were because the person was not at home at the time of vaccination or was out of town. In a few cases, the vaccine team missed the household, accounting for 53 missed doses. Refusing to take vaccine was not reported.

Reported AEFIs. Overall, 71 individuals of 1,274 respondents (5.6%) reported an AEFI (Table 3). Determining a causal relation between the vaccination and the reported symptoms was not attempted. [Insert Table 3]

Most AEFIs were considered mild or moderate, but 8 (0.6%) persons reported an AEFI as severe. Most (60%) of the persons reporting an AEFI did not seek treatment including 60% of those reporting a severe AEFI. 29.6% of the reported adverse events occurred in the first round, 40.9% in the second round and 29.6% in both rounds. The most common symptoms were abdominal pain (15), diarrhoea, (9), fever, nausea, and headache (each 6) reports. Table 4

For peer review only - http://bmjopen.bmj.com/site/about/guidelines.xhtml

provides additional information on the AEFIs. The reported AEFIs were infrequent relative to the number of doses distributed and there were no serious adverse events reported. [Insert Table4]

Community knowledge of oral cholera vaccines.

A majority (77%) of the respondents understood that vaccine was one of the ways to prevent cholera. There was a statistically significant association between education level and knowledge about OCV with those having at least a primary school education being almost twice as likely to know the number of required doses as compared to those with no education (OR 1.90, 95% CI 1.06, 3.44 (P = 0.03).

Sub-study Two, staff survey. A total of 242 and 125 staff responded to the first and second knowledge and practice (KP) surveys (KP1 and KP2). Most respondents were vaccination team members (89% and 87% in vaccine rounds 1 and 2, respectively). Almost all the respondents were knowledgeable about the cause of cholera, the importance of safe water in cholera prevention and the vaccine target group, but were less knowledgeable regarding potential adverse events following administration (AEFI) or how to advise vaccinees, with 29% and 16% being less informed about AEFI during the first and second surveys.

When staff were asked to suggest areas that needed improvement in future OCV campaigns, more than 10% suggested more timely payment of allowances, more time to sensitize and inform the communities on the benefits of the vaccine, and better transportation and facilitation allowances (payments to health workers to cover the cost they incurred when administering the vaccines or conducting activities related to the OCV campaign). Other suggestions included use of both static and mobile vaccination points, provision of gumboots, umbrellas, and more areas for vaccine storage in sub-counties where vaccine would be more accessible, more workers for hard to reach areas, and an increase in the number of vaccine days to complete the vaccinations and increase coverage.

DISCUSSION

The results of this monitoring and evaluation exercise documented important findings on the OCV campaign, the knowledge and practices of both the community and the health staff involved in the campaign and implications for the conduct of future OCV campaigns as part of an integrated cholera control strategy. These findings suggest that the OCV campaign in Hoima successfully provided the vaccine to a very large proportion of the target population in Hoima district, western Uganda. Approximately 93.6% of respondents reported receiving at least one

For peer review only - http://bmjopen.bmj.com/site/about/guidelines.xhtml

dose and 78.3% reported receiving two doses among residents. Given the mobile and transient nature of this population, this was noteworthy, and suggests that even better coverage may be possible for more settled populations in Uganda.

Since this was the first such campaign with OCV, there was concern that the population might be reluctant to accept it. This is a vaccine with which they were not familiar, it was given orally to all ages rather by injection to children, and two doses were required. Despite these potential constraints, we found that most people accepted taking the vaccine readily; however, some were not at home resulting in missed vaccinations.

High vaccination coverage is especially important when one is attempting to achieve herd protection. Since it is estimated that herd protection can be achieved with a coverage even lower than 90%;[19] the high coverage achieved in this campaign would be expected to induce significant indirect protection even among those who did not receive vaccine.[19 20]

It was noted in the administrative report from the MoH and during a stakeholder's meeting that one of the reasons for the reduction in the coverage during the second OCV dose was the unpredictable campaign dates for the second round. The vaccine for the second round had to be shipped and cleared through customs, and the timing for this was not certain. To avoid this problem in the future, a mechanism needs to be established to provide a better timeline for receipt of the vaccine shipments.

Community Reception to OCV. As with previous OCV campaigns outside Uganda, very few AEFI were reported.[21 22] Most of adverse events were considered mild or moderate and were self-limited. Despite the low prevalence of AEFI, the survey exposed the need to better inform the community about seeking treatment for more severe adverse events or for those that do not quickly resolve. This was especially true for families with little education who were less likely to seek medical attention for severe AEFI (data not shown). Notably, members in the community demonstrated good understanding of the rationale for the vaccine; however, a key takeaway from the survey was a need to better communicate the number of required doses, given that those with more education were twice as likely than those with no education to know the number of doses needed.

For peer review only - http://bmjopen.bmj.com/site/about/guidelines.xhtml

Staff Reception to OCV. Inclusion of staff KP survey contributed to the success of the project by identifying gaps among the staff knowledge and performance. Questioning the vaccine staff about their training and their experience in the field is not a common activity when conducting monitoring and evaluation activities during OCV campaigns. Many people had to be mobilized quickly and these were the key people who interacted with the communities. It was important that the staff accurately represent the campaign as an integrated cholera prevention program, but this was the first time these people carried out this role. The MoH felt it important to monitor their knowledge and behaviors as well as any constraints they felt in carrying out their functions. While they were generally knowledgeable about the disease and about the vaccine, these staff needed additional training regarding recognizing and managing AEFIs. They also faced challenges regarding logistical support. After the first staff KP survey, these gaps were communicated to the MoH so that appropriate actions could be taken to ensure that these gaps were addressed prior to the second round.

Most other OCV campaigns have also reported high coverage rates. These have included reports from Bangladesh[23 24], Malawi[25 26], Mozambique[21],Democratic Republic of Congo [27], Zambia [28], South Sudan [29 30], Iraq[31], Haiti[32], and Guinea [33]. Clearly, OCV is well accepted among these very diverse population groups where the vaccine campaigns have been carried out.

Important limitations of this study need to be mentioned. Ideally, one would prefer to conduct community studies prior to a campaign to understand knowledge and attitudes about cholera to improve communications regarding the upcoming campaign as part of an integrated strategy to control cholera. However, since the campaign was carried out on an emergency basis during an outbreak, a study prior to the campaign was not possible. Secondly, a community survey immediately after the first round might have provided feedback to the teams that would have improved the coverage for the second round. It should also be noted that a single informant provided information about receipt of the vaccine for all members of the household, so this informant might have incorrect information concerning one or more members of the household; however, since the vaccine was directly given to the household members together, it seems that inaccuracies would be minimal. It should be noted that we were not able to adjust for cluster sampling in the community surveys. If we have adjusted for cluster effect, it would have increased the variance slightly, but it would not affect the means.[34] The community KP survey did not include questions on attitudes regarding cholera. Since the survey had to be carried out

For peer review only - http://bmjopen.bmj.com/site/about/guidelines.xhtml

very quickly following the campaign, and since the survey was targeted to identify issues that would be immediately relevant to campaign performance, it was felt that understanding attitudes regarding cholera, even though important, would have required other qualitative methods requiring more time than was available. Similarly, direct observation of the training and coordination meetings would have been useful to independently assess the efficiency and effectiveness of these training and coordination meetings. Furthermore, there was no list of all workers in the campaign and many of the workers who participated in the second round had left prior to administering the questionnaire; thus, there were fewer respondents in the second round and the proportion of all workers who participated could not be determined precisely. Finally, it was not possible, given the time constraints, to fully integrate WASH interventions together with the OCV campaign, or to monitor and evaluate community and staff responsiveness to such integration.

In this outbreak 2,122 cholera cases and 44 deaths were reported, nearly all before the OCV campaign and over half occurred in the first two weeks of the outbreak. Of note, the outbreak started in February 2018 at about the same time the application for preventive use of OCV was being was submitted. The original application proposed a series of preventive campaigns over the next year, and Hoima, as well as neighbouring districts in western Uganda, were targeted for vaccination in the first round of these preventive campaigns. However, when the outbreak was identified, plans were quickly shifted so that an emergency campaign could be implemented to control the outbreak. Even though this emergency response was planned as quickly as possible, in fact, the outbreak was essentially over before the vaccine campaign could start, so it had no impact on the outbreak itself, but likely reduced the risk for future outbreaks.

Though the outbreak started with the influx of the refugees from DRC into Uganda, it quickly spread to the refugee host communities in Hoima. Therefore, to prevent rapid spread, improvement of cholera prevention measures for both the refugees and the host communities is paramount during resettlement.

CONCLUSION

This study suggests that the OCV campaign in Hoima district to prevent cholera was successful and achieved a high level of coverage in this population at high risk. However, there was need

For peer review only - http://bmjopen.bmj.com/site/about/guidelines.xhtml

to devote more effort on community sensitisation on the benefits of vaccination, as well as improving some logistic support during the campaign.

While a rapid response to this outbreak was appropriate, in fact, even with a rapidly organized campaign, the outbreak was over before the vaccine could be given; thus, the vaccine had no impact on this outbreak. Nevertheless, this area had already been identified as a hotspot, and it would have been targeted if the planned preventive campaign had proceeded as originally planned. Planners must realize that an area identified as a hotspot might experience an outbreak while preparations are underway for a preventive campaign and take this into account when allocating vaccine for preventive vs emergency campaigns. Since these are areas where cholera risk is high, outbreaks are likely to occur in these areas if there are delays in implementing preventive campaigns.

For peer review only - http://bmjopen.bmj.com/site/about/guidelines.xhtml

FIGURE LEGENDS

Figure 1. Epicurve of the Hoima Outbreak, 2018 with events identified in response to the outbreak

Figure 2: Map of Hoima District, showing sub-counties that received OCV and households where interviews were conducted (red dots)

For peer review only

460

Table 1. Age and Sex Breakdown of the Participants in the Cluster Survey

Age Group (years)	Male	Female	Total
**1 to 4**	98	93	191
**5 to 14**	236	207	443
**15 to 44**	210	285	495
**45+**	80	65	145
**Total**	625	650	1274

461

462

Table 2: Vaccination coverage post OCV campaign, Hoima District, Uganda, 2018.

**Total surveyed = 1274**	Round 1	Round 2	Received only one dose	Received two doses	Received at least one dose
**Reported number (%) (95% CI)**	1,164 (91.4) (89.7-92.8)	1,027 (80.6) (78.3-82.7)	195 (15.3) (13.4-17.4)	998 (78.3) (76.0-80.5)	1193 (93.6) (92.2-94.9)
**Confirmed by availability of the vaccination card (%) (95% CI)**	1,065 (83.6) (81.5-85.5)	823 (64.6) (61.9-67.2)	142 (11.1) (9.5-13.0)	792 (62.2) (59.5-64.8)	934 (73.3) (70.8-75.7)

463

464

Table 3: Treatment and Resolution of Adverse Events Following Immunization in Hoima District, Uganda, 2018.

**Symptoms**	Treatment		Status		
	**No treatment (%)**	**Treated (%)**	**Recovered (%)**	**Ongoing (%)**	**Improved, not to baseline (%)**
**Mild**	24(80.0)	6(20.0)	29(96.7)	1(3.3)	0(0.0)
**Moderate**	11(39.3)	17(60.7)	23(82.1)	3(10.7)	2(7.1)
**Severe**	8(61.5)	5(38.5)	10(76.9)	0(0.0)	3(23.1)

465

For peer review only - http://bmjopen.bmj.com/site/about/guidelines.xhtml

466

467 **Table 4. Onset and frequency of symptoms reported as adverse events**

	<6 hours	6-12 hours	12-24 hours	1-7 days	8-14 days	Total
**Diarrhoea**	3	2	2	2	0	9
**Vomiting**	3	0	0	2	0	5
**Nausea**	6	0	0	0	0	6
**Abdominal pain**	15*	0	0	0	0	15
**Stomach gurgling**	0	3	0	1	0	4
**Mouth ulcers**	0	0	0	0	1	1
**Cough**	1	1	0	3	1	6
**Felt feverish**	1	1	0	6	1	9
**Poor appetite**	1	0	0	0	0	1
**Dizziness**	0	3	0	0	0	3
**Fainted**	0	1	0	0	0	1
**Itching**	0	0	0	0	1	1
**Weakness**	0	0	0	1	0	1
**Headache**	3	0	2	1	0	6
**Other**	1	0	0	2	0	3
**Total**	34	11	4	18	4	71

- **Three persons reported abdominal pain in one household**

488

For peer review only

Acknowledgements

The authors thank the persons and institutions who supported Hoima district OCV monitoring and evaluation study. Special thanks are extended to the research assistants who dedicated their time to meticulously collect the data and the persons who collaborated in the preparation of the different key documents used in this study. We thank the staff, the communities in the study area and the local leadership of Hoima districts for the cooperation during this study. We are grateful to Ambrose Buyinza Wabwire for technical support with drawing of the maps.

We thank the Ministry of Health leadership for guidance; the Office of the Prime Minister (OPM) for the permission to access data on the refugee population. We are grateful to the following institutions and bodies; Ministry of Health, Makerere University School of Public Health, World Health Organisation, UNICEF and UNHCR for technical guidance.

Footnotes

Contributors: Conceived and designed the study: GB, MR, WAB, ABS, CGO, DAS. Discussed, critically revised and approved the study protocol: GB, MR, WAB, CGO, DAS. Performed the research: GB, MR, WAB, AO, FR, IA. Analysed the data: GB, MR, WAB, AO, DAS. Wrote the first draft: GB, MR. Wrote the final DAS. Elaborated, discussed and approved the final version: GB, MR, AB, ABS, AO, FR, IA, CGO, and DAS.

Funding: Funding for the study was provided by the Bill and Melinda Gates Foundation (OPP1148763) which provided financial support through the John Hopkins University, Delivery of Oral Cholera Vaccine Effectively (DOVE) project. The funding agency had no role in collecting, analysing or interpreting the results.

Declaration of the conflict of interest

The authors have no conflict of interest to declare.

Additional data sharing: Data are available upon reasonable request

For peer review only

REFERENCES

1. Clemens JD, Nair GB, Ahmed T, et al. Cholera. *Lancet* 2017 doi: 10.1016/S0140-6736(17)30559-7
2. Cholera vaccines: WHO position paper - August 2017. *Wkly Epidemiol Rec* 2017;92(34):477-98.
3. Kirigia JM, Sambo LG, Yokouide A, et al. Economic burden of cholera in the WHO African region. *BMC Int Health Hum Rights* 2009;9:8. doi: 10.1186/1472-698X-9-8
4. Lessler J, Moore SM, Luquero FJ, et al. Mapping the burden of cholera in sub-Saharan Africa and implications for control: an analysis of data across geographical scales. *Lancet* 2018;391(10133):1908-15. doi: 10.1016/S0140-6736(17)33050-7
5. Bwire G, Ali M, Sack DA, et al. Identifying cholera "hotspots" in Uganda: An analysis of cholera surveillance data from 2011 to 2016. *PLoS Negl Trop Dis* 2017;11(12):e0006118. doi: 10.1371/journal.pntd.0006118
6. Cummings MJ, Wamala JF, Eyura M, et al. A cholera outbreak among semi-nomadic pastoralists in northeastern Uganda: epidemiology and interventions. *Epidemiology and infection* 2012;140(8):1376-85. doi: 10.1017/S0950268811001956 [published Online First: 2011/09/29]
7. Bwire G, Mwesawina M, Baluku Y, et al. Cross-Border Cholera Outbreaks in Sub-Saharan Africa, the Mystery behind the Silent Illness: What Needs to Be Done? *PLoS One* 2016;11(6):e0156674. doi: 10.1371/journal.pone.0156674
8. Bwire G, Malimbo M, Maskery B, et al. The burden of cholera in Uganda. *PLoS Negl Trop Dis* 2013;7(12):e2545. doi: 10.1371/journal.pntd.0002545
9. Oguttu DW, Okullo A, Bwire G, et al. Cholera outbreak caused by drinking lake water contaminated with human faeces in Kaiso Village, Hoima District, Western Uganda, October 2015. *Infect Dis Poverty* 2017;6(1):146. doi: 10.1186/s40249-017-0359-2
10. Alajo SO, Nakavuma J, Erume J. Cholera in endemic districts in Uganda during El Nino rains: 2002-2003. *Afr Health Sci* 2006;6(2):93-7. doi: 10.5555/afhs.2006.6.2.93 [published Online First: 2006/08/19]
11. Montgomery M, Jones MW, Kabole I, et al. No end to cholera without basic water, sanitation and hygiene. *Bull World Health Organ* 2018;96(6):371-71A. doi: 10.2471/BLT.18.213678
12. Legros D, Partners of the Global Task Force on Cholera C. Global Cholera Epidemiology: Opportunities to Reduce the Burden of Cholera by 2030. *J Infect Dis* 2018 doi: 10.1093/infdis/jiy486
13. Deployments from the oral cholera vaccine stockpile, 2013-2017. *Wkly Epidemiol Rec* 2017;92(32):437-42.
14. Odevall L, Hong D, Digilio L, et al. The Euvichol story - Development and licensure of a safe, effective and affordable oral cholera vaccine through global public private partnerships. *Vaccine* 2018;36(45):6606-14. doi: 10.1016/j.vaccine.2018.09.026
15. Bwire G, Munier A, Ouedraogo I, et al. Epidemiology of cholera outbreaks and socio-economic characteristics of the communities in the fishing villages of Uganda: 2011-2015. *PLoS Negl Trop Dis* 2017;11(3):e0005407. doi: 10.1371/journal.pntd.0005407

For peer review only - http://bmjopen.bmj.com/site/about/guidelines.xhtml

16. Uganda_Bureau_of_Statistics_(UBOS). Demographic and Health Survey. 2016 Kampala and Rockville Maryland2016 [Available from: https://www.dhsprogram.com/pubs/pdf/FR333/FR333.pdf accessed Sept 18. 2020 2020.

17. Henderson RH, Sundaresan T. Cluster sampling to assess immunization coverage: a review of experience with a simplified sampling method. *Bull World Health Organ* 1982;60(2):253-60.

18. Food_and_Drug_Administration. Guidance for Industry. Toxicity Grading Scale for Healthy Adult and Adolescent Volunteers Enrolled in Preventive Vaccine Clinical Trials Rockville, Maryland USA: Food and Drug Administration, Center for Biologics Evaluation and Research 2007 [cited 2020. Available from: https://www.fda.gov/media/73679/download accessed September 22 2020.

19. Longini IM, Jr., Nizam A, Ali M, et al. Controlling endemic cholera with oral vaccines. *PLoS medicine* 2007;4(11):e336. doi: 10.1371/journal.pmed.0040336 [published Online First: 2007/11/30]

20. Ali M, Emch M, von Seidlein L, et al. Herd immunity conferred by killed oral cholera vaccines in Bangladesh: a reanalysis. *Lancet* 2005;366(9479):44-9. doi: S0140-6736(05)66550-6 [pii]

10.1016/S0140-6736(05)66550-6 [published Online First: 2005/07/05]

21. Sema Baltazar C, Rafael F, Langa JPM, et al. Oral cholera vaccine coverage during a preventive door-to-door mass vaccination campaign in Nampula, Mozambique. *PLoS One* 2018;13(10):e0198592. doi: 10.1371/journal.pone.0198592

22. Phares CR, Date K, Travers P, et al. Mass vaccination with a two-dose oral cholera vaccine in a long-standing refugee camp, Thailand. *Vaccine* 2016;34(1):128-33. doi: 10.1016/j.vaccine.2015.10.112

23. Khan AI, Khan IA, Siddique SA, et al. Feasibility, coverage and cost of oral cholera vaccination conducted by icddr,b using the existing national immunization service delivery mechanism in rural setting Keraniganj, Bangladesh. *Hum Vaccin Immunother* 2019;15(6):1302-09. doi: 10.1080/21645515.2018.1528833

24. Uddin MJ, Wahed T, Saha NC, et al. Coverage and acceptability of cholera vaccine among high-risk population of urban Dhaka, Bangladesh. *Vaccine* 2014;32(43):5690-5. doi: 10.1016/j.vaccine.2014.08.021

25. Grandesso F, Rafael F, Chipeta S, et al. Oral cholera vaccination in hard-to-reach communities, Lake Chilwa, Malawi. *Bull World Health Organ* 2018;96(12):817-25. doi: 10.2471/BLT.17.206417

26. Sauvageot D, Saussier C, Gobeze A, et al. Oral cholera vaccine coverage in hard-to-reach fishermen communities after two mass Campaigns, Malawi, 2016. *Vaccine* 2017;35(38):5194-200. doi: 10.1016/j.vaccine.2017.07.104

27. Massing LA, Aboubakar S, Blake A, et al. Highly targeted cholera vaccination campaigns in urban setting are feasible: The experience in Kalemie, Democratic Republic of Congo. *PLoS Negl Trop Dis* 2018;12(5):e0006369. doi: 10.1371/journal.pntd.0006369

For peer review only

For peer review only - http://bmjopen.bmj.com/site/about/guidelines.xhtml

28. Poncin M, Zulu G, Voute C, et al. Implementation research: reactive mass vaccination with single-dose oral cholera vaccine, Zambia. *Bull World Health Organ* 2018;96(2):86-93. doi: 10.2471/BLT.16.189241
29. Parker LA, Rumunu J, Jamet C, et al. Neighborhood-targeted and case-triggered use of a single dose of oral cholera vaccine in an urban setting: Feasibility and vaccine coverage. *PLoS Negl Trop Dis* 2017;11(6):e0005652. doi: 10.1371/journal.pntd.0005652
30. Porta MI, Lenglet A, de Weerdt S, et al. Feasibility of a preventive mass vaccination campaign with two doses of oral cholera vaccine during a humanitarian emergency in South Sudan. *Trans R Soc Trop Med Hyg* 2014;108(12):810-5. doi: 10.1093/trstmh/tru153
31. Lam E, Al-Tamimi W, Russell SP, et al. Oral Cholera Vaccine Coverage during an Outbreak and Humanitarian Crisis, Iraq, 2015. *Emerg Infect Dis* 2017;23(1):38-45. doi: 10.3201/eid2301.160881
32. Tohme RA, Francois J, Wannemuehler K, et al. Oral Cholera Vaccine Coverage, Barriers to Vaccination, and Adverse Events following Vaccination, Haiti, 2013. *Emerg Infect Dis* 2015;21(6):984-91. doi: 10.3201/eid2106.141797
33. Luquero FJ, Grout L, Ciglenecki I, et al. First outbreak response using an oral cholera vaccine in Africa: vaccine coverage, acceptability and surveillance of adverse events, Guinea, 2012. *PLoS Negl Trop Dis* 2013;7(10):e2465. doi: 10.1371/journal.pntd.0002465
34. Population_Survey_Analysis. Methods: Complex sampling: populationsurveyanaysis.com; [Available from: http://www.populationsurveyanalysis.com/wp-content/uploads/2014/09/complexsampling_handout.pdf accessed November 17, 2020 2020.

For peer review only

For peer review only - http://bmjopen.bmj.com/site/about/guidelines.xhtml

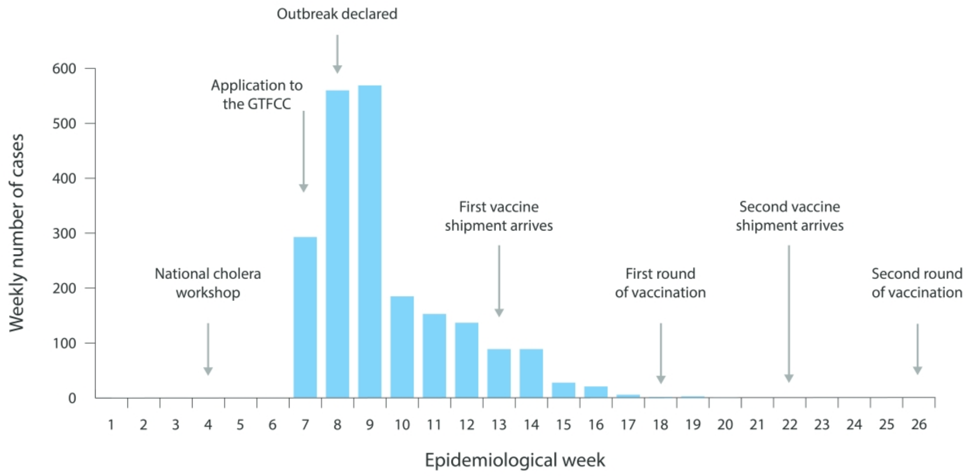

Figure 1. Epicurve of the Hoima Outbreak, 2018 with events identified in response to the outbreak

542x305mm (300 x 300 DPI)

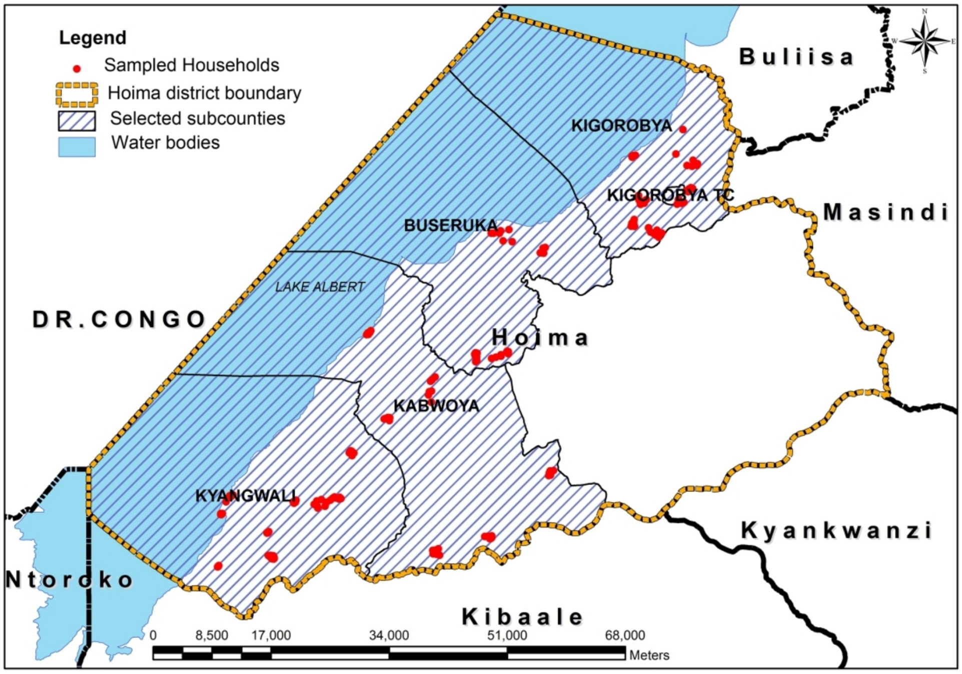

Figure 2: Map of Hoima District, showing sub-counties that received OCV and households where interviews were conducted (red dots)

170x119mm (300 x 300 DPI)
